# GABA_A_ Receptor Modulators with a Pyrazolo[1,5-a]quinazoline Core: Synthesis, Molecular Modelling Studies and Electrophysiological Assays

**DOI:** 10.3390/ijms232113032

**Published:** 2022-10-27

**Authors:** Letizia Crocetti, Gabriella Guerrini, Fabrizio Melani, Claudia Vergelli, Maria Paola Mascia, Maria Paola Giovannoni

**Affiliations:** 1 Neurofarba, Pharmaceutical and Nutraceutical Section, University of Florence, Via Ugo Schiff 6, 50019 Sesto Fiorentino, Italy; 2 CNR-Institute of Neuroscience, Cagliari, Cittadella Universitaria, 09042 Monserrato, Italy

**Keywords:** GABA_A_ receptor modulators, molecular modeling, pyrazolo[1,5-a]quinazoline, electrophysiological studies

## Abstract

As a continuation of our study in the GABA_A_ receptor modulators field, we report the design and synthesis of new 8-chloropyrazolo[1,5-a]quinazoline derivatives. Molecular docking studies and the evaluation of the ‘Proximity Frequencies’ (exploiting our reported model) were performed on all the final compounds (**3**, **4**, **6a**–**c**, **7a**,**b**, **8**, **9**, **12a**–**c**, **13a**,**b**, **14**–**19**) to predict their profile on the α1β2γ2-GABA_A_R subtype. Furthermore, to verify whether the information coming from this virtual model was valid and, at the same time, to complete the study on this series, we evaluated the effects of compounds (1–100 µM) on the modulation of GABA_A_ receptor function through electrophysiological techniques on recombinant α1β2γ2L-GABA_A_ receptors expressed in *Xenopus laevis* oocytes. The matching between the virtual prediction and the electrophysiological tests makes our model a useful tool for the study of GABA_A_ receptor modulators.

## 1. Introduction

The neurotransmitter α-aminobutyric acid, GABA, interacts with two different types of receptors, GABA_A_ and GABA_B_ receptors (GABA_A_Rs, GABA_B_R), which belong to the heteropentameric Ligand Gated Ion Channel (LGCI) superfamily with Cys-loop topology and are responsible for fast neuronal inhibition. The receptor is formed by five protein subunits arranged to form an anion channel permeable to chloride ions, which causes the postsynaptic hyperpolarization and the following inhibition of signal transmission after the binding of the neurotransmitter.

Sequences of six α (1–6), three β (1–3), three γ (1–3), one δ, three ρ (1–3), one ε, π and θ subunits could assemble the pentameric channel, even if most GABA_A_Rs contain α-, β- and γ-subunits with stoichiometry 2α, 2β and 1γ. The α1β2γ2-GABA_A_R subtypes constitute the most significant population of receptors, followed by α2β2γ2-, α3β2γ2- and α5β2γ2-GABA_A_Rs. However, other combinations of GABA_A_R containing different isoforms are distributed in the CNS and even located extrasynaptically (GABA_A_ receptors with α4, α6 and δ subunits) [1]. The neurotransmitter binds the two orthosteric sites at the interface of the α−/β+ subunits, while the benzodiazepine binding site is between α-/γ+ subunits; moreover, recently, the presence of another ‘benzodiazepine binding site’ at the α+/β− interface (the so-called low-affinity diazepam binding site) [2] has been confirmed [3] and defined as the “pyrazoloquinolinones binding site” [4].

In recent years, cryogenic electron microscopy (cryo-EM) has made it possible to better elucidate the fine structure of synaptic or extrasynaptic GABA_A_Rs, as well as the mechanism and the possible binding modes of benzodiazepine ligands. These results also highlight the amino acid residues involved in the agonists (diazepam, alprazolam) or antagonists (flumazenil) binding and help to rationalize the design and synthesis of new benzodiazepine site ligands [3,5,6].

As a continuation of our research on ‘benzodiazepine receptor ligands’ with pyrazoloquinazoline (PQ) scaffold [7,8], we report here the synthesis of new 8-chloropyrazolo[1,5-a]quinazoline derivatives. Therefore, using the “Proximity Frequencies” [9], a molecular dynamic model (MD model) and statistical analysis developed in our laboratory, we tried to predict the pharmacological GABA_A_ modulator profile (agonist or antagonist) for all new compounds. Finally, to test/verify the reliability of the MD results, all new compounds have been tested in electrophysiological studies on recombinant GABA_A_R (α1β2γ2-GABA_A_R), expressed in *Xenopus Laevis* oocytes by evaluating the variation of produced chlorine current.

## 2. Results and Discussion

### 2.1. Chemistry

**Scheme 1:**
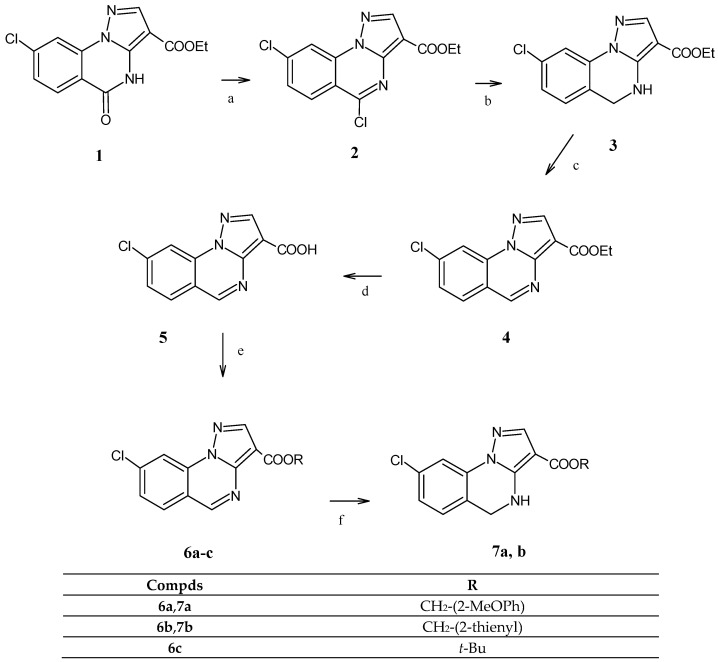
Reagents and conditions: (a) POCl_3_/PCl_5_, reflux temperature; (b) NaBH_4_, CH_2_Cl_2_/EtOH abs., r.t.; (c) Toluene, Pd/C 10%, reflux temperature; (d) 15% NaOH solution, 80 °C then HCl; (e) SOCl_2_; CH_2_Cl_2,_ suitable alcohol; (f) NaCNBH_3_, AcOH, refluxed temperature.

**Scheme 2:**
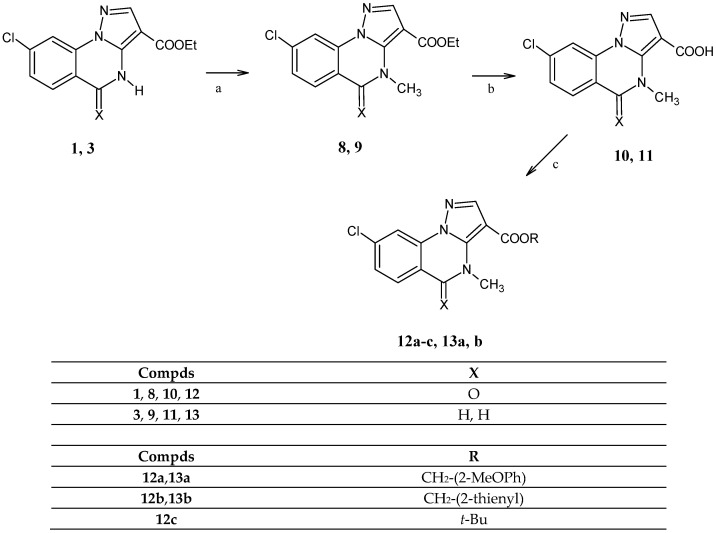
Reagents and conditions: (a) DMF/K_2_CO_3_/MeI for **8**; *t*BuOH/NaH/MeI for **9**; (b) 10% NaOH solution, reflux temperature, then HCl; (c) SOCl_2_; CH_2_Cl_2_, suitable alcohol.

**Scheme 3:**
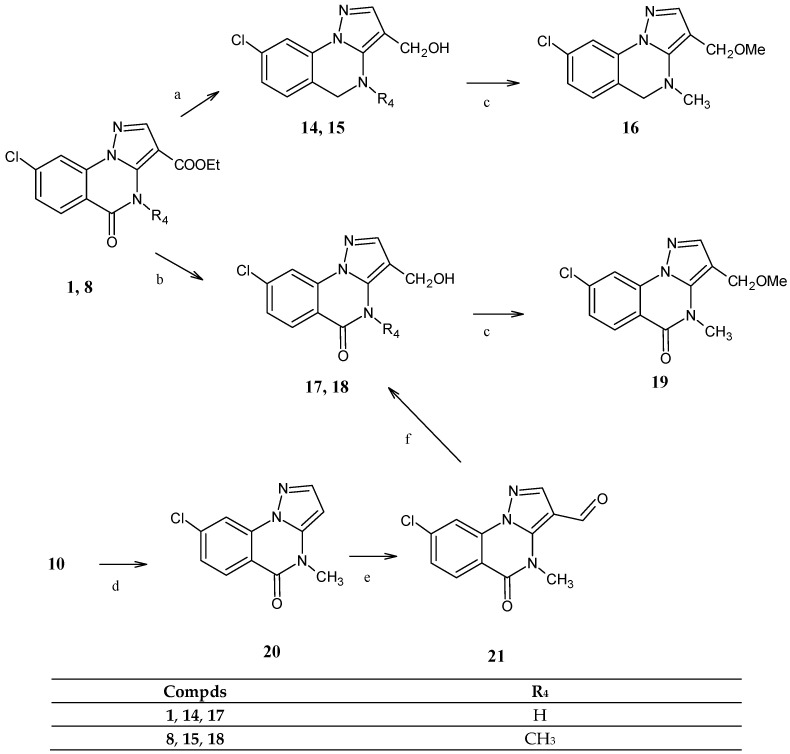
Reagents and conditions: (a) tBuOH/NaBH_4_, 17 days for **14** and 36 h for **15**; (b) THF/LiBH_4_/MeOH, reflux temperature; (c) DMSO/KOH/MeI; (d) HCl reflux temperature; (e) HMTA/AcOH reflux temperature; (f) THF/MeOH, NaBH_4_, r.t.

The chemical steps to obtain the final compounds, the 3-ester (**6a**–**c**, **7a**,**b**, **12a**–**c**, **13a**,**b**), the hydroxymethyl (**14**, **15**, **17**, **18**) and the 3-methoxymethyl derivatives (**16** and **19**), are depicted in Scheme 1, Scheme 2 and Scheme 3 and the NMR spectra of some representative compounds are reported in Supplementary Materials.

To obtain the final 8-chloropyrazolo[1,5-a]quinazolines and 8-chloro-5-oxo- 4,5-dihydropyrazolo[1,5-a]quinazolines of type **6** and **7**, respectively (Scheme 1), the starting product, ethyl 8-chloro-5-oxo-4,5-dihydropyrazolo[1,5-a]quinazoline-3- carboxylate [10] **1**, was reacted as previously reported by us [7] and afforded the ethyl 5,8-dichloropyrazolo[1,5-a]quinazoline-3-carboxylate **2**. The latter undergoes reduction at 5-position (5-C-Cl) in sodium borohydride/ethanol/DCM, giving in a good yield the corresponding 4,5-dihydroderivative **3**, which by 4,5-dehydrogenation performed in toluene and Pd/C gave the heteroaromatic derivative **4**, ethyl 8-chloropyrazolo[1,5-a] quinazoline-3-carboxylate. The next hydrolysis (NaOH and then HCl) furnished the carboxylic acid **5**, which in turn was transformed into 3-acyl chloride (not isolated), useful for the synthesis of the final desired esters (**6a**–**c**) by using the suitable alcohol (2-methoxybenzyl alcohol, 2-thiophenmethanol and *t*-butanol, respectively). Finally, the treatment of **6a** and **6b** with NaCNBH_4_/AcOH gave the final 4,5-dihydro-3-ester derivatives **7a**,**b**.

To obtain the esters of type **12** and **13**, bearing a methyl group at the nitrogen at position 4 of the 5-oxo-4,5-dihydropyrazolo[1,5-a]quinazoline and 4,5-dihydropyrazolo[1,5-a]quinazoline scaffold, respectively (Scheme 2), the starting products **1** and **3**, already seen in Scheme 1, were alkylated at 4-position with MeI in DMF/K_2_CO_3_ or *t*-BuOH/NaH, obtaining compounds **8** [10] and **9**, respectively. Again, the transformation into the corresponding carboxylic acids **10** [10] and **11** was performed, and the next treatment with thionyl chloride/DCM and the suitable alcohol (2-methoxybenzyl alcohol, 2-thiophenmethanol and *t*-butanol) gave the 8-chloro-4-methyl-5-oxo-4,5-dihydro pyrazolo[1,5-a]quinazoline 3-esters **12a**–**c**, and the 8-chloro-4-methyl-4,5-dihydropyrazolo[1,5-a]quinazoline 3-esters **13a**,**b**.

Scheme 3 depicts the synthetic pathways to obtain the 3-hydroxymethyl and the 3-methoxymethyl derivatives in both 4,5-dihydropyrazolo[1,5-a]quinazoline (**14**–**16**) and 5-oxo-4,5-dihydropyrazolo[1,5-a]quinazoline (**17**–**19**) scaffolds. The starting materials **1** and **8** [10], depending on the type of the reducing agent, can undergo total (lactamic functions and ester group) or partial (only ester group) reduction. In particular, a total reduction occurred with NaBH_4_/t-BuOH, albeit at different times, affording 8-chloro-4,5-dihydropyrazolo[1,5-a]quinazoline-3-methanol (**14**) and 8-chloro-4-methyl- -4,5-dihydropyrazolo[1,5-a]quinazoline-3-methanol (**15**), respectively. With NaBH_4_/THF/methanol, the selective 3-ester reduction to the 3-hydroxymethyl group occurred, affording compounds **17** and **18**, the latter in very low yield.

An alternative route to obtain compound **18** with a better yield is depicted at the bottom of the Scheme: starting from **10 [10]**, already seen in Scheme 2, a decarboxylation in HCl 12M was performed, obtaining compound **20**, which, treated with HMTA (hexamethylenetetramine), gave the corresponding 3-carboxyaldeide derivative **21** that was quickly reduced (20 min, room temperature) to hydroxymethyl group with NaBH_4_/THF/methanol. Finally, all the 3-hydroxymethyl derivatives (**14**–**18**) were subjected to alkylation, achieving the final compounds 8-chloro-3-methoxymethyl-4-methyl- -4,5-dihydropyrazolo[1,5-a]quinazoline **16** and 8-chloro-3-methoxymethyl-4-methyl pyrazolo[1,5-a]quinazolin-5(4H)-one **19**.

### 2.2. Molecular Dynamic Studies

A molecular docking study and an evaluation of the ‘Proximity Frequencies’ [9] were performed on all new final compounds (**3**, **4**, **6a**–**c**, **7a**–**b**, **8**, **9**, **12a**–**c**, **13a**, **b**, **14**–**19**) to predict their profile on the α1β2γ2-GABA_A_R subtype.

The value of Proximity Frequencies (PFs), used in a linear discriminant function (LDA), was able to correctly collocate 70.6% of agonists and 72.7% of antagonists by combining a double PF (αVal203-γThr142) with a triple PF (αHis102-αTyr160-γTyr58). The predictive capacity was evaluated on an appropriate training set of molecules with a cross-validation ‘leave one out’ (LOO) procedure.

The agonist compounds, during a molecular dynamic simulation (60 ns), were simultaneously close to the αVal203 and γThr142 amino acids, with a frequency of 37% compared to the frequency of 16% found by the antagonist compounds, while the antagonist compounds were simultaneously close to the αHis102, αTyr160 and γTyr58 amino acids, with a frequency of 35% against a frequency of 13% for agonist compounds. Moreover, it highlighted that agonists’ orientation in the binding site is significantly different from that assumed by the antagonists, as reported in the literature [11].

All the 3D structures of the molecules, as a training set and new final compounds, were designed (DS ViewerPro 6.0 Accelrys Software Inc., San Diego, CA, USA) and placed in the binding site of the BDZs with the AUTODOCK 4.2 (Morris et al. 2009) docking program. The structure of the BDZ binding site was obtained from the recently solved GABA_A_R structure (PDB ID 6D6T) [3].

The docking program performed on twenty compounds (**3**, **4**, **6a**–**c**, **7a**–**b**, **8**, **9**, **12a**–**c**, **13a**, **14**, **15**, **16**, **17**, **18** and **19**) gave a cluster of conformation(s) for each compound (rmsd 2.0). The evaluation of trajectories in the dynamic simulation was performed on the conformations that covered at least 90% of poses; thus, for compounds **3**, **9**, **15**, **16**, **17** and **19**, it was acceptable to use one conformation, while two conformations for all other compounds (**4**, **6a**–**c**, **7a**–**b**, **8**, **12a**–**c**, **13a**, **14**, and **18**) were required. The molecular dynamic simulations were performed on an isolated portion of the protein between the α and γ chains comprising all amino acids within a radius of 2 nm from the center of the benzodiazepine binding site. Applying the PF model [9] to the new synthesized compounds, we obtained the results reported in Table 1.

**Figure 1:**
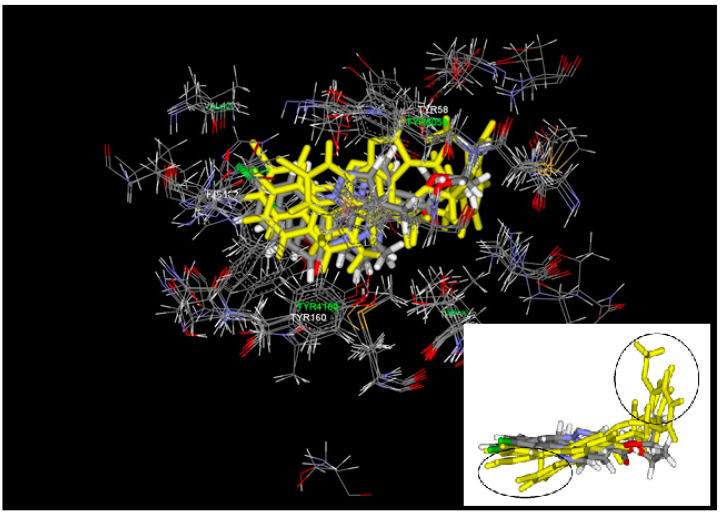
Superimposition of antagonists (colored by element) and agonists (yellow). The black circles evidence the different areas occupied by agonists.

From prediction based on the discriminant function calculated in the model [9], compounds **4**, **6c**, **7b** and **12a** gave uncertain results, since the prediction for the different conformations disagreed, while compounds **6b**, **8**, **13b** and **14**–**16** showed borderline results, with about 50% of collocation for the two classes. Derivatives **6a**, **7a**, **12b**, **c**, **13a**, **17** and **18** are collocated in the agonist class, and among them, **6a**, **13a** and **18** reach a percentage of prediction of 93.1%, 72.4% and 64%, respectively. On the other hand, compounds **3**, **9** and **19** are collocated in the antagonist class, with a percentage prediction range of 62–73%. From molecular dynamic studies, the poses of predicted antagonists (**3**, **9** and 1**9**) and agonists (**6a**, **13a** and **18**) in the binding site emerged (Figure 1), but their overlapping seems to suggest that the two groups of compounds have the same orientation, in contrast with what we evidenced in the training set used to build the model. Therefore, the different predicted profiles could be justified by occupying other areas in the binding site (black circles in Figure 1).

For example, the 3-hydroxymethyl **18** and the 3-methoxymethyl derivative **19**, predicted agonist and antagonist, respectively, interact with a different frequency and strength in the considered amino acid area during the molecular dynamic simulation.

**Figure 2:**
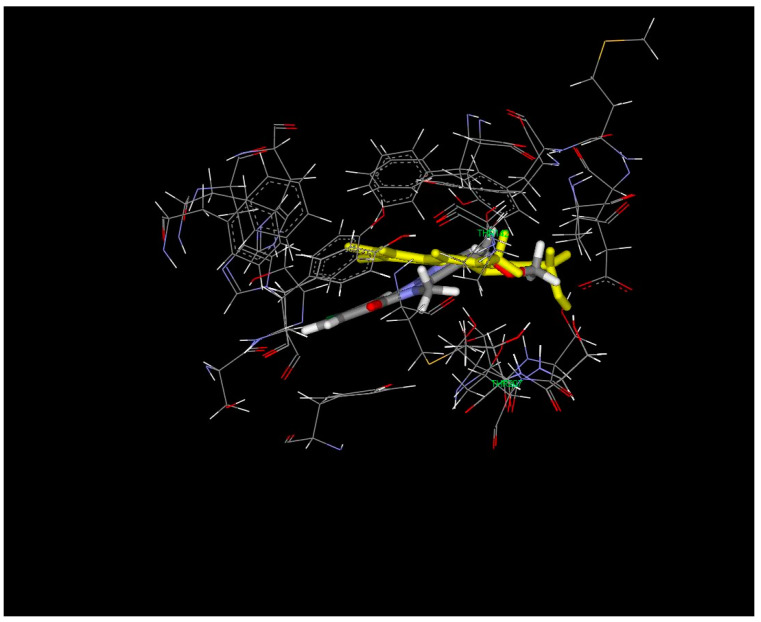
Superimposition of compounds **18** (yellow) and **19**.

Figure 2 reports the overlapping of the two compounds **18** and **19** and the different orientations in the selected region.

This different accommodation in the binding site of **18** (agonist) with respect to **19** (antagonist) could be due to hydrogen bond interactions with the amino acids in the region of αThr142 and αThr207.

**Figure 3:**
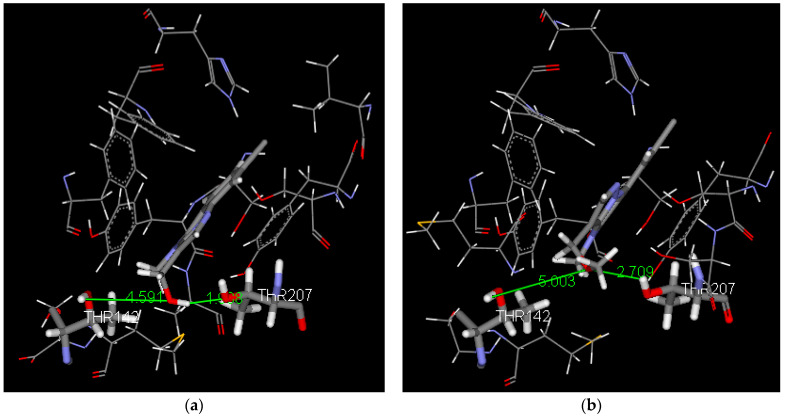
Hydrogen bond interaction of compound **18** (**a**) and of compound **19** (**b**) in the last frame of the dynamic simulation.

**Figure 4:**
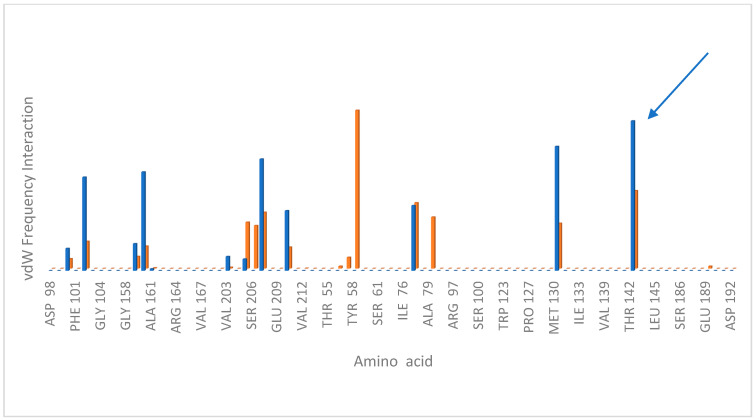
Frequency contacts of Van der Waals interaction measured during the dynamic simulation of the ligand–receptor complex; compound **18** (agonist), blue column, compound **19** (antagonist), orange column.

Considering only a hydrogen bond distance (D-H^…^A) < 3 Å, the predicted agonist **18** forms a hydrogen bond interaction with αThr207 at an average distance of 1.9 Å (sd 0.2), with a frequency of 52%. On the contrary, the predicted antagonist **19** interacts more weakly with the same residue, with a longer average distance of 2.4 Å (sd 0.2) and a frequency < 8% (see Figure 3). Regarding the Van der Waals interactions, considering only those with a distance < 3 Å, the interaction with γTyr58 is exclusive for compound **19** (predicted antagonist), while compound **18** interacts with a more significant frequency with γTyr142, as can be seen from the histogram in Figure 4.

**Figure 5:**
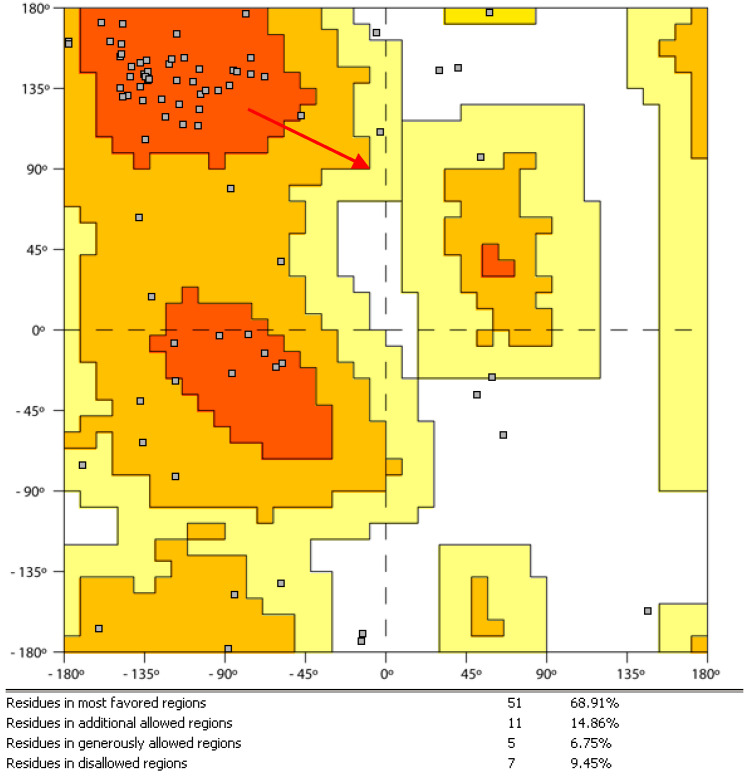
Ramachandran plot of the protein-ligand complex conformation at the end of 60 ns.

The molecular dynamic simulation of the protein–ligand complex was realized on a portion of protein identified by amino acids located at a distance < 2.0 nM from the center of the ‘benzodiazepine binding site’. During the simulation, the selected fragment did not show excessive distortion, as evidenced by Ramachandran’s plot [12] (Figure 5), which is relative to the complex conformation at the end of the 60 ns of simulation: only 9% of amino acid residues are located in a disallowed region (white).

### 2.3. Biological Evaluation

**Figure 6:**
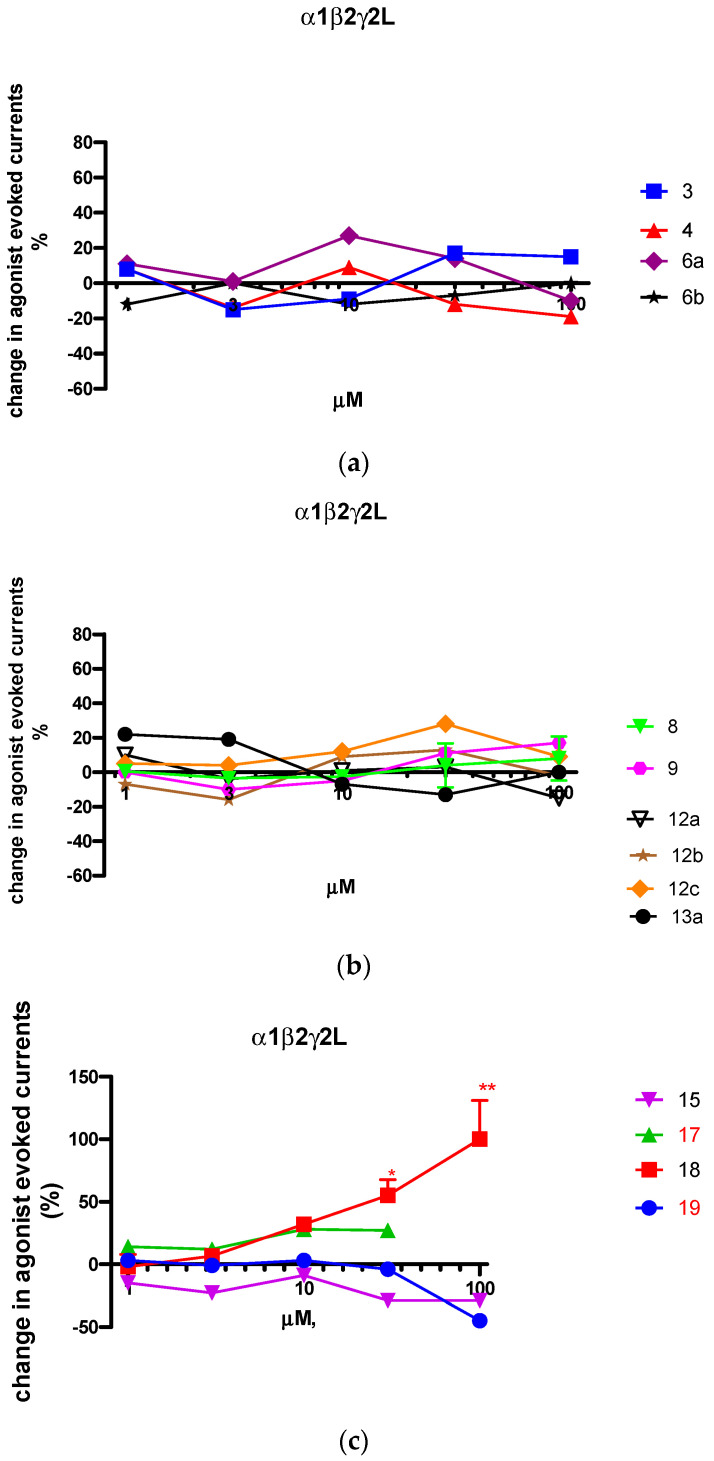
Concentration-response curves of compounds **3**, **4**, **6a**, **b**, **8**, **9**, **12a**–**c**, **13a** (panel (**a**,**b**)) and **15**, **17**–**19** (panel (**c**)) on GABA-induced Cl^−^ currents in *Xenopus laevis* oocytes expressing recombinant α1β2γ2L-GABA_A_ receptors. Data are expressed as the percentage modulation of the response induced by GABA at EC_5-10_ values (approximatively 3–5 μM) and are the mean ± SEM of values obtained from two to nine oocytes.* *p* < 0.05; ** *p* < 0.01.

In order to verify whether the information coming from this virtual model is valid and, at the same time, to complete the study on this series, as the next step of our research, we evaluated the profile of the new compounds through electrophysiological techniques on recombinant α1β2γ2L-GABA_A_ receptors expressed in *Xenopus laevis* oocytes, and the effects of compounds (1-100 µM) on the modulation of GABA_A_ receptor function were assessed (see Figure 6). The two sections of this figure, respectively, report the experiments performed on 3-esterderivatives **3**, **4**, **6a**, **b**, **8**, **9**, **12a**–**c**, **13a** (Figure 6a,b) and on 3-hydroxymethyl and 3-methoxymethyl derivatives **15**, **17**–**19** (Figure 6c).

**Figure 7:**
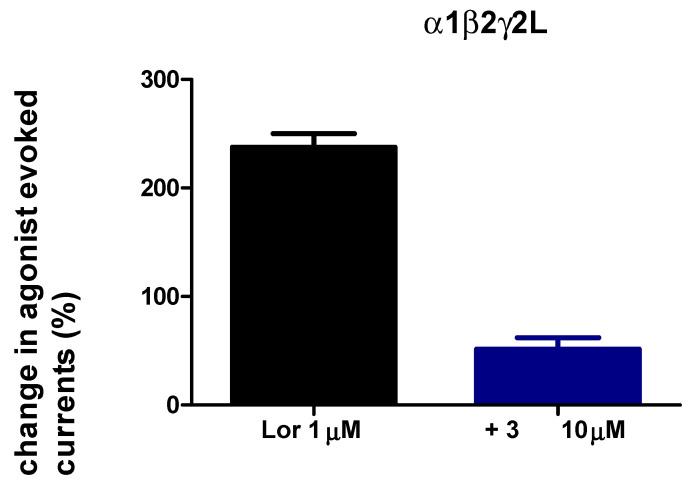
Compound **3** antagonized the potentiation of GABA-induced Cl^−^ currents by lorazepam in *Xenopus laevis* oocytes expressing recombinant α1β2γ2L-GABA_A_ receptors. Data are expressed as the percentage modulation of the response induced by GABA at EC_5-10_ values (approximatively 3–5 μM) and are the mean ± SEM of values obtained from four oocytes.

As evident in Figure 6a, all 3-ester derivatives were not able to modulate the GABA_A_ function, and we can hypothesize that they act as null modulators. To confirm that these compounds act at the benzodiazepine binding site, the representative compound **3** was evaluated for its ability to antagonize the full agonist lorazepam (1 µM). Figure 7 clearly demonstrates that this compound can abolish the chlorine current evoked by the agonist used as a standard, thus confirming its antagonist profile.

**Figure 8:**
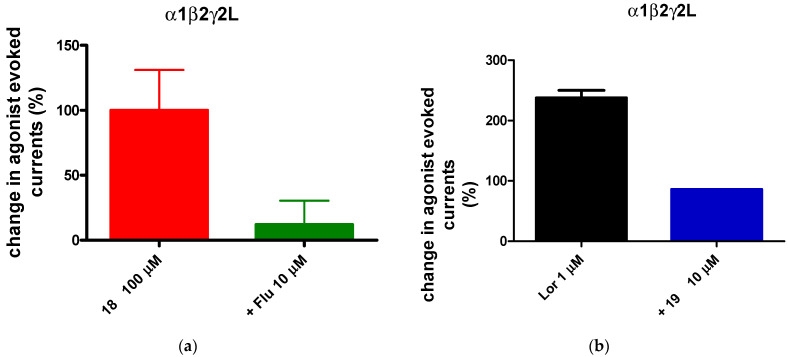
(**a**) Compound **18** antagonized by flumazenil; (**b**) lorazepam antagonized by **19**.

Very intriguing results emerged from derivatives bearing a hydroxymethyl or methoxymethyl group at position 3 of the scaffold (Figure 6b). In particular, the 8-chloro-3-hydroxymethyl-4-methylpyrazolo[1,5-a]quinazoline-5-one **18** behaves as an agonist, significantly enhancing the chlorine current at 100 μM (E_max_ + 100%). On the other hand, the 8-chloro-3-(methoxymethyl)-4-methylpyrazolo[1,5-a]quinazoline-5-one **19**, which differs from **18** for the presence of the methoxymethyl chain at position 3, acts as a null modulator, thus indicating that the -OH group is important for agonist activity. These two compounds were tested in the presence of the antagonist flumazenil (compound **18)** and of the agonist lorazepam (compound **19**) (Figure 8a,b) to evaluate whether they act through the benzodiazepine binding site. Figure 8a reports flumazenil’s ability to antagonize **18** (100 µM), and Figure 8b shows the capacity of **19** to revert the agonist effect of lorazepam, in both cases confirming that the two compounds bind at the benzodiazepine binding site.

## 3. Materials and Methods

Reagents and starting materials were obtained from commercial sources. Extracts were dried over Na_2_SO_4_, and the solvents were removed under reduced pressure. All reactions were monitored by thin layer chromatography (TLC) using commercial plates pre-coated with Merck silica gel 60 F-254. Visualization was performed by UV fluorescence (λ_max_ = 254 nm) or by staining with iodine or potassium permanganate. Chromatographic separations were performed on a silica gel column by gravity chromatography (Kieselgel 40, 0.063–0.200 mm; Merck, Kenilworth, NJ, USA), flash chromatography (Kieselgel 40, 0.040–0.063 mm; Merck) and silica gel preparative TLC (Kieselgel 60 F_254_, 20 × 20 cm, 2 mm). Yields refer to chromatographically and spectroscopically pure compounds, unless otherwise stated. Compounds were named following IUPAC rules, as applied by Beilstein-Institut AutoNom 2000 (4.01.305) or CA Index Name. All melting points were determined on a hot-stage Büchi microscope and are uncorrected. The identity and purity of intermediates and final compounds were ascertained through ^1^H-NMR, ^13^C-NMR and TLC chromatography. Monodimensional spectra ^1^H-NMR and ^13^C-NMR were registered by a 400 MHz field through Avance 400 apparatus (Bruker Biospin Version 002 with SGU). Chemical shifts (d) are in parts per million (ppm) approximated by the nearest 0.01 ppm, using the solvent as internal standard. Coupling constants (J) are in Hz; they were calculated by Top Spin 3.1 and approximated by 0.1 Hz. Data are reported as follows: chemical shift, multiplicity (exch, exchange; br, broad; s, singlet; d, doublet; t, triplet; q, quartet; m, multiplet; or a combination of those, e.g., dd), integral, assignments and coupling constant. Mass spectra (m/z) were recorded on an ESI-MS triple quadrupole (Varian 1200 L) system, in positive ion mode, by infusing a 10 mg/L solution of each analyte dissolved in a mixture of mQ H_2_O:acetonitrile 1:1 *v*/*v*. All new compounds possess a purity ≥95%; microanalyses indicated by the symbols of the elements were performed with a Perkine-Elmer 260 elemental analyzer for C, H and N, and they were within ±0.4% of the theoretical values.

### 3.1. Chemistry

**Ethyl 5**,**8-dichloropyrazolo[1**,**5-a]quinazoline-3-carboxylate(2).** The starting material, ethyl 8-chloro-5-oxo-4,5-dihydropyrazolo[1,5-a]quinazoline-3-carboxylate [10] (0.6 mmol), was solubilized in 3 mL of POCl_3_ and PCl_5_ (75 mg) and maintained at refluxing temperature for 2 h, monitoring the reaction by TLC. Then the solution was evaporated to dryness, and the residue was treated with ice/water, obtaining a slightly yellow residue that was filtered. The raw material was pure enough for the next reaction. Recrystallization solvent: ethanol; yield 66%; mp 178–181 °C. TLC: toluene/ethyl acetate/acetic acid 8:2:2 *v*/*v*/*v*; IR ν cm^−1^ 1718, 1597, 1270, 1057; ^1^H-NMR (400 MHz, DMSO-d_6_) δ 8.61 (s, 1H, H-2); 8.46 (d, 1H, H-9, *J =* 2.0 Hz); 8.33 (d, 1H, H-6, *J =* 8.8 Hz); 7.84 (dd, 1H, H-7, *J_1_ =* 8.8 Hz, *J_2_ =* 2.0 Hz); 4.31 (q, 2H, CH_2_, *J =* 7.2 Hz); 1.31 (t, 3H, CH_3_, *J =* 7.2 Hz). ESI-MS calcd for C_13_H_9_N_3_O_2_Cl_2_ (309.01); found: 311.00 *m*/*z* [M + H]^+^.Anal. C_13_H_9_N_3_O_2_Cl_2_ (C, H, N).

**Ethyl 8-chloro-4**,**5-dihydropyrazolo[1**,**5-a]quinazoline-3-carboxylate (3).** ethyl 5,8-dichloropyrazolo[1,5-a]quinazoline-3-carboxylate **2** (0.1 g, 0.32 mmol) was dissolved In a solution of ethanol (12 mL) and dichlorometane (7 mL) and sodium borohydride (0.12 g, 3.14 mmol) was added in small portions. The reaction was maintained at room temperature for 30′, and the disappearance of the starting material was monitored by TLC. The evaporation of solvent to dryness gave a residue that was recovered with ethanol 80%, filtered and recrystallized by ethanol, yield 75%, cream crystals, mp 182–183 °C. TLC: toluene/ethyl acetate/methanol 8:2:1.5 *v*/*v*/*v*; IR ν cm^−1^ 3100, 1718, 1270, 1057; ^1^H-NMR (400 MHz, CDCl_3_) δ 7.71 (s, 1H, H-2); 7.25 (s, 1H, H-9); 7.11 (dd, 1H, H-7, *J_1_ =* 8.0 Hz, *J_2_ =* 2.0 Hz); 7.04 (d, 1H, H-6, *J =* 8.0 Hz); 5.94 (exch br s, 1H, NH); 4.61 (s, 2H, CH_2_); 4.28 (q, 2H, CH_2_, *J =* 6.8 Hz); 1.33 (t, 3H, CH_3_, *J =* 6.8 Hz). ^13^C-NMR (100 MHz, DMSO-d_6_) δ 162.86, 148.78, 142.59, 135.50, 133.20, 129.08, 125.59, 113.72, 94.90, 42.33, 14.96. ESI-MS calcd for C_13_H_12_N_3_O_2_Cl (277.71); found: 279.06 *m*/*z* [M + H]^+^. Anal. C_13_H_12_N_3_O_2_Cl (C, H, N).

**Ethyl 8-chloropyrazolo[1**,**5-a]quinazoline-3-carboxylate (4).** ethyl 8-chloro-4,5-dihydropyrazolo[1,5-a]quinazoline-3-carboxylate **3** (0.1 g, 0.36 mmol) was dissolved in 25 mL of toluene and Pd/C 10% (0.03 g) was added. The reaction was refluxed for 24 h and then the catalyst was filtered off. The solution was evaporated to dryness and the white residue was recovered with water, filtered and recrystallized by ethanol, yield 57%; mp 170–172 °C. TLC: toluene/ethyl acetate/methanol 8:2:1.5 *v*/*v*/*v*; IR ν cm^−1^ 1718, 1270, 1057; ^1^H-NMR (400 MHz, CDCl_3_) δ 9.13 (s, 1H, H-5); 8.53 (m 2H, H-2 and H-9); 7.97 (d, 1H, H-6, *J =* 8.4 Hz); 7.59 (d, 1H, H-7, *J =* 8.0 Hz); 4.45 (q, 2H, CH_2_, *J =* 6.8 Hz); 1.48 (t, 3H, CH_3_, *J =* 6.8 Hz). ^13^C-NMR (100 MHz, DMSO-d_6_) δ 162.89, 155.54, 144.54, 142.32, 134.71, 133.45, 129.08, 125.98, 119.87, 114.72, 113.70, 94.98, 59.52, 14.16. ESI-MS calcd for C_13_H_10_N_3_O_2_Cl (275.69); found: 277.04 *m*/*z* [M + H]^+^. Anal. C_13_H_10_N_3_O_2_Cl (C, H, N).

**8-Chloropyrazolo[1**,**5-a]quinazoline-3-carboxylic acid (5).** The ester **4** (0.1 g, 0.36 mmol) was dissolved in a few drops of diglyme and then 10 mL of 15% sodium hydroxide solution was added and refluxed for 2.5 h. The final suspension was diluted with water, acidified with HCl conc. until pH = 1, and the precipitate recovered by filtration was pure enough for the next step. Recrystallized solvent: ethanol, yield 78%; mp 277–280 °C. TLC: toluene/ethyl acetate/acetic acid 8:2:1 *v*/*v*/*v*; IR ν cm^−1^ 3475, 1685; ^1^H-NMR (400 MHz, DMSO-d_6_) δ 12.62 (exch br s, 1H, OH); 9.33 (s, 1H, H-5); 8.57 (s, 1H, H-2); 8.41 (s, 1H, H-9); 8.33 (d, 1H, H-6, *J =* 8.4 Hz); 7.80 (d, 1H, H-7, *J =* 8.8 Hz). ESI-MS calcd for C_11_H_6_N_3_O_2_Cl (247.64); found: 249.01 *m*/*z* [M + H]^+^. Anal. C_11_H_6_N_3_O_2_Cl (C, H, N).

**General procedure for obtaining compounds 6a–c.** The carboxylic acid **5** (0.5 mmol) was transformed into the corresponding 3-carbonyl chloride by reaction with an excess of SOCl_2_ in anhydrous conditions. After the standard work-up, the residue was suspended in dichloromethane (6 mL), and the suitable alcohol (excess, 0.15 mL) was added; TLC monitored the reaction until the disappearance of the starting material. Then the final solution was evaporated to dryness, and the residue recuperated with isopropyl ether and recrystallized.

**2-Methoxybenzyl 8-chloropyrazolo[1**,**5-a]quinazoline-3-carboxylate (6a).** From **4** and methoxybenzyl alcohol; white crystals, recrystallized by ethanol, yield 59%; mp 223–226 °C. TLC: toluene/ethyl acetate/methanol 8:2:1.5 *v*/*v*/*v*; IR ν cm^−1^ 1722, 1626, 1277, 1057; ^1^H-NMR (400 MHz, DMSO-d_6_) δ 9.32 (s, 1H, H-5); 8.54 (s, 1H, H-2); 8.36 (s, 1H, H-9); 8.30 (d, 1H, H-7, *J =* 8.4 Hz); 7.76 (d, 1H, Ph, *J =* 7.2 Hz); 7.29–7.35 (m, 2H, H-6 and Ph); 6.99 (d, 1H, Ph, *J =* 8.4 Hz); 6.90 (t, 1H, Ph, *J =* 7.2 Hz); 4.67 (s, 2H, OCH_2_); 3.80 (s, 3H, OCH_3_). ^13^C-NMR (100 MHz, DMSO-d_6_) δ163.53, 155.32, 146.13, 144.95, 140.48, 136.27, 136.27, 131.93, 131.21, 130.83, 128.70, 127.52, 125.72, 120.85, 117.89, 114.54, 111.71, 108.80, 56.01, 42.18. ESI-MS calcd for C_19_H_14_N_3_O_3_Cl (367.79); found: 369.07 *m*/*z* [M + H]^+^. Anal. C_19_H_14_N_3_O_3_Cl (C, H, N).

**Thiophen-2-yl-methyl 8-chloropyrazolo[1**,**5-a]quinazoline-3-carboxylate (6b).** From **4** and 2-thiophenmethanol; white crystals, recrystallized by isopropanol, yield 45%; mp 229–232 °C. TLC: toluene/ethyl acetate/methanol 8:2:1.5 *v*/*v*/*v*; IR ν cm^−1^ 1730, 1626, 1277, 1057; ^1^H-NMR (400 MHz, DMSO-d_6_) δ 9.22 (s, 1H, H-5); 8.22 (s, 1H, H-2); 8.16 (d, 1H, H-6, *J =* 8.4 Hz); 8.09 (d, 1H, H-9, *J =* 2.0 Hz); 7.60 (dd, 1H, H-7, *J_1_ =* 8.4 Hz, *J_2_ =* 2.0 Hz); 7.57 (d, 1H, H-5 Thiophene, *J =* 5.2 Hz); 7.24 (d, 1H, H-3 Thiophene, *J =* 2.8 Hz); 7.03 (dd, 1H, H-4 Thiophene, *J_1_ =* 5.2 Hz, *J_2_ =* 2.8 Hz); 5.46 (s, 2H, OCH_2_). ^13^C-NMR (100 MHz, DMSO-d_6_) δ 163.56, 160.41, 155.38, 146.17, 140.51, 131.98, 129.54, 127.56, 123.50, 117.88, 114.59, 108.12, 63.15. ESI-MS calcd for C_16_H_10_N_3_O_2_SCl (343.79); found: 345.02 *m*/*z* [M + H]^+^. Anal. C_16_H_10_N_3_O_2_SCl (C, H, N).

***tert*-Butyl 8-chloropyrazolo[1**,**5-a]quinazoline-3-carboxylate (6c).** From **4** and *tert*-butanol; white crystals, recrystallized by isopropanol, yield 48%; mp 228–231 °C. TLC: toluene/ethyl acetate/methanol 8:2:1.5 *v*/*v*/*v*; IR ν cm^−1^ 1730, 1626, 1277, 1057; ^1^H-NMR (400 MHz, DMSO-d_6_) δ 9.34 (s, 1H, H-5); 8.56 (s, 1H, H-2); 8.40 (d, 1H, H-9, *J =* 2.0 Hz); 8.33 (d, 1H, H-6, *J =* 8.6 Hz); 7.79 (d, 1H, H-7, *J =* 8.6 Hz); 2.47 (s, 9H, (CH_3_)_3_). ^13^C-NMR (100 MHz, DMSO-d_6_) δ 164.32, 155.52, 146.15, 139.54, 131.97, 130.34, 127.56, 125.06, 124.76, 123.65, 114.58, 108.23, 84.12, 28.78. ESI-MS calcd for C_15_H_14_N_3_O_2_Cl (303.75); found: 305.07 *m*/*z* [M + H]^+^. Anal. C_15_H_14_N_3_O_2_Cl (C, H, N).

**General procedure for obtaining compounds 7a-b.** The starting esters **6a** and **6b** (0.240 mmol) were solubilized in glacial acetic acid under nitrogen flow. Then, 0.972 mmol of sodium cyanoborohydride (NaBH_3_CN) was added, and the reaction was refluxed for 1 h and monitored by TLC. The final solution was cooled at room temperature and water added until a precipitate was formed; the raw product was purified by recrystallization by a suitable solvent.

**2-Methoxybenzyl 8-chloro-4**,**5-dihydropyrazolo[1**,**5-a]quinazoline-3-carboxylate (7a).** White crystals, recrystallized by isopropanol, yield 50%; mp 168–170 °C. TLC: toluene/ethyl acetate/methanol 8:2:1 *v*/*v*/*v*; IR ν cm^−1^ 1722, 1626, 1277, 1057; ^1^H-NMR (400 MHz, CD_3_CN-d_3_) δ 8.18 (m, 4H, H-2, H-9, H-7 and H-6); 7.50 (d, 1H, Ph, *J =* 8.0 Hz); 7.44 (d, 1H, Ph, *J =* 8.0 Hz); 7.21 (d, 1H, Ph, *J =* 8.4 Hz); 7.04 (t, 1H, Ph, *J =* 7.2 Hz); 5.46 (s, 2H, OCH_2_); 3.86 (s, 5H, OCH_3_, CH_2_NH). ^13^C-NMR (100 MHz, DMSO-d_6_) δ 166.45, 157.14, 156.82, 141.06, 138.99, 133.98, 132.77, 131.88, 131.43, 131.12, 130.56, 130.29, 129.45, 128.90, 116.48, 114.58, 65.98, 56.42, 55.08. ESI-MS calcd for C_19_H_16_N_3_O_3_Cl (369.81); found: 371.09 *m*/*z* [M + H]^+^. Anal. C_19_H_16_N_3_O_3_Cl (C, H, N).

**Thiophen-2-yl-methyl 8-chloro-4**,**5-dihydropyrazolo[1**,**5-a]quinazoline-3-carboxylate(7b).** White crystals, recrystallized by isopropanol, yield 45%; mp 229–232 °C. TLC: toluene/ethyl acetate/methanol 8:2:1.5 *v*/*v*/*v*; IR ν cm^−1^ 1725, 1630, 1277, 1057; ^1^H-NMR (400 MHz, CD_3_CN-d_3_) δ 8.16 (d, 1H, H-6, *J =* 8.6 Hz); 8.15 (s, 1H, H-2); 8.14 (d, 1H, H-9, *J =* 2.0 Hz); 7.50 (dd, 1H, H-7, *J_1_ =* 8.6 Hz, *J_2_ =* 2.0 Hz); 7.44 (dd, 1H, H-5 Thiophene, *J_1_ =* 5.2 Hz, *J_2_ =* 1.2 Hz); 7.21 (d, 1H, H-3 Thiophene, *J =* 3.2 Hz); 7.03 (dd, 1H, H-4 Thiophene, *J_1_ =* 5.2 Hz, *J_2_ =* 3.2 Hz); 5.46 (s, 2H, OCH_2_); 3.85 (s, 2H, CH_2_NH). ^13^C-NMR (100 MHz, CD_3_CN-d_3_) δ 167.14, 155.23, 154.21, 140.06, 139.43, 138.18, 137.41, 135.53, 125.86, 124.63, 122.06, 119.74, 116.48, 114.32, 61.63, 58.47, 56.41. ESI-MS calcd for C_16_H_12_N_3_O_2_SCl (345.79); found: 347.03 *m*/*z* [M + H]^+^. Anal. C_16_H_12_N_3_O_2_SCl (C, H, N).

**Ethyl-8-chloro-4-methyl-5-oxo-4**,**5-dihydropyrazolo[1**,**5-a]quinazoline-3-carboxylate (8).** A suspension of **1** (0.34 mmoles) in DMF abs. (2.5 mL) and anhydrous potassium carbonate (0.34 mmoles) was maintained for 15 min under stirring at room temperature; then, methyl iodide (0.34 mmoles, 0.04 mL) was added and the reaction was heated at 80° C, monitored by TLC. After the starting material disappeared, water/ice was added and the precipitate was filtered under suction and purified by recrystallization with ethanol. White crystals, yield 90 %; mp 159–161 °C. TLC: toluene/ethyl acetate/methanol 8:2:1.5 *v*/*v*/*v*; ^1^H-NMR (400 MHz, CDCl_3_) δ 8.27 (s, 1H, H-2); 8.18 (d, 1H, H-6, *J =* 8.4 Hz); 8.12 (d, 1H, H-9, *J =* 1.2 Hz); 7.62 (dd, 1H, H-7, *J_1_ =* 8.0 Hz, *J_2_ =* 1.6 Hz); 4.26 (q, 2H, CH_2_, *J =* 7.0 Hz); 3.80 (s, 3H, NCH_3_); 1.31 (t, 3H, CH_3_, *J =* 7.0 Hz). ESI-MS calcd for C_14_H_12_N_3_O_3_Cl (305.72); found: 307.05 *m*/*z* [M + H]^+^. Anal. C_14_H_12_N_3_O_3_Cl (C, H, N). [10]

**Ethyl 8-chloro-4-methyl-4**,**5-dihydropyrazolo[1**,**5-a]quinazoline-3-carboxylate (9).** A suspension of **3** (0.35 mmoles) in *tert*-butanol (4.0 mL) and a 60% sodium hydride dispersion in mineral oil (2.92 mmoles) was maintained for 15 min under stirring at room temperature; then, methyl iodide (0.36 mmoles, 0.05 mL) was added and the reaction was refluxed, monitoring the reaction by TLC. After the starting material disappeared, water/ice and HCl 1.5N were added, and the aqueous phase was extracted with ethyl acetate. After the normal work-up, the residue was recovered with ethanol and recrystallized by the same solvent. White crystals, yield 38%; mp 152–154 °C. TLC: toluene/ethyl acetate/methanol 8:2:1.5 *v*/*v*/*v*; IR ν cm^−1^ 3475, 1685; ^1^H-NMR (400 MHz, CDCl_3_) δ 7.80 (s, 1H, H-2); 7.50 (d, 1H, H-6, *J =* 8.0 Hz); 7.39–7.42 (m, 2H, H-9, H-7); 4.45 (s, 2H, CH_2_N); 4.30 (q, 2H, CH_2_, *J =* 7.2 Hz); 2.52 (s, 3H, NCH_3_); 1.38 (t, 3H, CH_3_, *J =* 7.2 Hz). ESI-MS calcd for C_14_H_14_N_3_O_2_Cl (291.74); found: 293.07 *m*/*z* [M + H]^+^. Anal. C_14_H_14_N_3_O_2_Cl (C, H, N).

**General procedure for the synthesis of 10 and 11.** A suspension of **8** or **9** (0.35 mmoles) in 10% sodium hydroxide solution (5 mL) was refluxed under stirring for 1 h, monitoring the reaction by TLC. After the starting material disappeared, water/ice and HCl 6N were added, and the precipitate was filtered under suction.

**8-Chloro-4-methyl-5-oxo-4**,**5-dihydropyrazolo[1**,**5-a]quinazoline-3-carboxylic acid (10).** From **8**; white crystals, yield 80%; mp 234–237 °C. TLC: toluene/ethyl acetate/methanol 8:2:1.5 *v*/*v*/*v*; ^1^H-NMR (400 MHz, DMSO-d_6_) δ 11.50 (exch br s, 1H, OH); 8.22 (s, 1H, H-2); 8.17 (d, 1H, H-6, *J =* 8.0 Hz); 8.09 (s, 1H, H-9); 7.59 (d, 1H, H-7, *J =* 8.0 Hz); 3.83 (s, 3H, NCH_3_). ESI-MS calcd for C_12_H_8_N_3_O_3_Cl (277.66); found: 279.02 *m*/*z* [M + H]^+^. Anal. C_12_H_8_N_3_O_3_Cl (C, H, N). [10]

**8-Chloro-4-methyl-4**,**5-dihydropyrazolo[1**,**5-a]quinazoline-3-carboxylic acid (11).** From **9**; white crystals, yield 40%; mp 160–162 °C. TLC: toluene/ethyl acetate/acetic acid 8:2:1.5 *v*/*v*/*v*; IR ν cm^−1^ 3475, 1685; ^1^H-NMR (400 MHz, DMSO-d_6_) δ 12.78 (exch br s, 1H, OH); 8.23 (s, 1H, H-2); 8.16 (d, 1H, H-6, *J =* 8.2 Hz); 8.09 (s, 1H, H-9); 7.60 (d, 1H, H-7, *J =* 8.0 Hz); 3.83 (s, 2H, CH_2_N); 2.47 (s, 3H, NCH_3_). ESI-MS calcd for C_12_H_10_N_3_O_2_Cl (263.68); found: 265.04 *m*/*z* [M + H]^+^.Anal. C_12_H_10_N_3_O_2_Cl (C, H, N).

**General procedure for obtaining compounds 12a-c and 13a**, **b.** The carboxylic acid **10** or **11** (0.5 mmol) was transformed into the corresponding 3-carbonyl chloride by reaction with an excess of SOCl_2_ in anhydrous conditions. After the standard work-up, the residue was suspended in dichloromethane (6 mL), and the suitable alcohol (excess, 0.15 mL) was added; TLC monitored the reaction until the disappearance of the starting material. Then, the final solution, after washing with 5% NaOH solution, was evaporated to dryness, and the residue recuperated with isopropyl ether or ethanol 80% and recrystallized.

**2-Methoxybenzyl-8-chloro-4-methyl-5-oxo-4**,**5-dihydropyrazolo[1**,**5-a]quinazoline-3-carboxylate (12a).** From **10** and methoxybenzyl alcohol; white crystals, recrystallized by *i*-propanol, yield 40%; mp 200–202 °C. TLC: toluene/ethyl acetate/acetic acid 3:1:1 *v*/*v*/*v*; IR ν cm^−1^ 1722, 1626, 1277, 1057; ^1^H-NMR (400 MHz, DMSO-d_6_) δ 8.24 (s, 1H, H-2); 8.17 (d, 1H, H-6, *J =* 8.0 Hz); 8.09 (s, 1H, H-9); 7.61 (d, 1H, H-7, *J =* 8.0 Hz); 7.40 (m, 1H, Ph); 7.33 (m, 1H, Ph); 7.06 (m, 1H, Ph); 6.96 (m, 1H, Ph); 5.27 (s, 2H, OCH_2_); 3.80 (s, 3H, OCH_3_); 3.76 (s, 3H, NCH_3_). ^13^C-NMR (100 MHz, DMSO-d_6_) δ 167.45, 164.24, 154.56, 143.75, 140.78, 139.45, 135.54, 130.94, 129.85, 120.85, 115.09, 111.44, 77.29, 62.07. ESI-MS calcd for C_20_H_16_N_3_O_4_Cl (397.82); found: 399.08 *m*/*z* [M + H]^+^. Anal. C_20_H_16_N_3_O_4_Cl (C, H, N).

**Thiphen-2-yl-methyl-8-chloro-4-methyl-5-oxo-4**,**5-dihydropyrazolo[1**,**5-a]quinazoline-3-carboxylate (12b).** From **10** and 2-thiophenmethanol; white crystals, recrystallized by isopropanol, yield 70%; mp 178–180 °C. TLC: toluene/ethyl acetate/acetic acid 3:1:1 *v*/*v*/*v*; IR ν cm^−1^ 1730, 1626, 1277, 1057; ^1^H-NMR (400 MHz, DMSO-d_6_) δ 8.22 (s, 1H, H-2); 8.16 (d, 1H, H-6, *J =* 8.4 Hz); 8.09 (d, 1H, H-9, *J =* 2.0 Hz); 7.60 (dd, 1H, H-7, *J_1_ =* 8.4 Hz, *J_2_ =* 2.0 Hz); 7.56 (d, 1H, H-5 Thiophene, *J =* 5.2 Hz); 7.24 (d, 1H, H-3 Thiophene, *J =* 2.8 Hz); 7.03 (dd, 1H, H-4 Thiophene, *J_1_ =* 5.2 Hz, *J_2_ =* 2.8 Hz); 5.46 (s, 2H, OCH_2_); 3.78 (s, 3H, NCH_3_). ^13^C-NMR (100 MHz, DMSO-d_6_) δ 164.43, 145.11, 130.93, 129.11, 128.07, 127.40, 127.24, 115.11, 60.92, 34.12. ESI-MS calcd for C_17_H_12_N_3_O_3_SCl (373.81); found: 375.03 *m*/*z* [M + H]^+^. Anal. C_17_H_12_N_3_O_3_SCl (C, H, N).

***tert*-Butyl-8-chloro-4-methyl-5-oxo-4**,**5-dihydropyrazolo[1**,**5-a]quinazoline-3-carboxylate (12c).** From **10** and *tert*-butanol; white crystals, recrystallized by isopropanol, yield 28%; mp 123–126 °C. TLC: toluene/ethyl acetate/acetic acid 3:1:1 *v*/*v*/*v*; IR ν cm^−1^ 1730, 1626, 1277, 1057; ^1^H-NMR (400 MHz, DMSO-d_6_) δ 8.18 (s, 1H, H-2); 8.16 (d, 1H, H-6, *J =* 8.8 Hz); 8.09 (d, 1H, H-9, *J =* 2.0 Hz); 7.59 (dd, 1H, H-7, *J_1_ =* 8.8 Hz, *J_2_ =* 2.0 Hz); 3.76 (s, 3H, NCH_3_); 1.51 (s, 9H, (CH_3_)_3_). ^13^C-NMR (100 MHz, DMSO-d_6_) δ 145.87, 130.94, 127.08, 115.08, 34.15, 28.36. ESI-MS calcd for C_16_H_16_N_3_O_3_Cl (333.77); found: 335.09 *m*/*z* [M + H]^+^. Anal. C_16_H_16_N_3_O_3_Cl (C, H, N).

**2-Methoxybenzyl-8-chloro-4-methyl-4**,**5-dihydropyrazolo[1**,**5-a]quinazoline-3-carboxylate (13a).** From **11** and methoxybenzyl alcohol; white crystals, recrystallized by ethanol 96%, yield 89%; mp 153–154 °C. TLC: toluene/ethyl acetate/methanol 8:2:1 *v*/*v*/*v*; IR ν cm^−1^ 1766, 1635, 1276; ^1^H-NMR (400 MHz, DMSO-d_6_) δ 8.32 (s, 1H, H-2); 8.25 (d, 1H, H-6, *J =* 8.0 Hz); 8.17 (s, 12H, H-9); 7.68 (d, 1H, H-7, *J =* 8.0 Hz); 7.48 (d, 1H, Ph, *J =* 8.0 Hz); 7.41 (d, 1H, Ph, *J =* 8.0 Hz); 7.13 (d, 1H, Ph, *J =* 8.4 Hz); 7.03 (t, 1H, Ph, *J =* 7.2 Hz); 5.34 (s, 2H, OCH_2_); 3.89 (s, 3H, OCH_3_); 3.84 (s, 2H, CH_2_N); 2.55 (s, 3H, NCH_3_). ^13^C-NMR (100 MHz, DMSO-d_6_) δ 165.90, 156.44, 141.13, 138.73, 127.38, 127.05, 125.93, 125.86, 125.29, 124.15, 65.30, 61.92, 56.15, 38.40. ESI-MS calcd for C_20_H_18_N_3_O_3_Cl (383.83); found: 385.10 *m*/*z* [M + H]^+^. Anal. C_20_H_18_N_3_O_3_Cl (C, H, N).

**Thiophen-2-yl-methyl 8-chloro-4-methyl-4**,**5-dihydropyrazolo[1**,**5-a]quinazoline-3-carboxylate (13b).** From **11** and 2-thiophenmethanol; white crystals, recrystallized by ethanol 96%, yield 50%; mp 155–156 °C. TLC: toluene/ethyl acetate/methanol 8:2:1.5 *v*/*v*/*v*; IR ν cm^−1^ 1730, 1626, 1277, 1057; ^1^H-NMR (400 MHz, DMSO-d_6_) δ 7.50 (d, 1H, H-6, *J =* 8.6 Hz); 8.15 (s, 1H, H-2); 8.14 (d, 1H, H-9, *J =* 2.0 Hz); 7.50 (dd, 1H, H-7, *J_1_ =* 8.6 Hz, *J_2_ =* 2.0 Hz); 7.44 (dd, 1H, H-5 Thiophene, *J_1_ =* 5.2 Hz, *J_2_ =* 1.2 Hz); 7.21 (d, 1H, H-3 Thiophene, *J =* 3.2 Hz); 7.03 (dd, 1H, H-4 Thiophene, *J_1_ =* 5.2 Hz, *J_2_ =* 3.2 Hz); 5.46 (s, 2H, OCH_2_); 3.85 (s, 2H, CH_2_NH). ^13^C-NMR (100 MHz, DMSO-d_6_) δ 165.50, 144.44, 143.48, 141.13, 139.83, 127.38, 127.00, 125.93, 125.86, 125.29, 125.19, 65.95, 63.89, 38.19. ESI-MS calcd for C_17_H_14_N_3_O_2_SCl (359.83); found: 361.05 *m*/*z* [M + H]^+^. Anal. C_17_H_14_N_3_O_2_SCl (C, H, N).

**General procedure for obtaining compounds 14**, **15.** The starting material **1** or **8** [10] (0.5 mmol) was dissolved in 10 mL of *t*-butanol abs. and 0.15 g (8.91 mmoles) of sodium borohydride was quickly added. The reaction was maintained at reflux temperature (36 h—days), then was worked up by adding water/HCl 1N (10/3 mL) and extracted with ethyl acetate. After it dried under anhydrous sodium sulphate, the evaporation of the organic layer gave a residue recovered with water and filtered.

**(8-Chloro-4**,**5-dihydropyrazolo[1**,**5-a]quinazoline-3-yl)methanol (14).** From **1** after 17 days; white crystals, recrystallized by ethanol 96%, yield 72%; mp 145–147 °C. TLC: toluene/ethyl acetate/acetic acid 8:2:1 *v*/*v*/*v*; IR ν cm^−1^ 3100; ^1^H-NMR (400 MHz, DMSO-d_6_) δ 11.76 (exch br s, 1H, OH); 8.06 (m, 2H, H-6 and H-2); 7.94 (s, 1H, H-9); 7.44 (d, 1H, H-7, *J =* 8.0 Hz); 6.07 (exch br s, 1H, NH); 3.70 (s, 2H, CH_2_N); 3.29 (s, 2H, CH_2_O). ^13^C-NMR (100 MHz, DMSO-d_6_) δ 148.41, 140.53, 138.69, 137.73, 135.92, 130.09, 129.28, 114.57, 108.74, 58.65, 54.41. ESI-MS calcd for C_11_H_10_N_3_OCl (235.67); found: 237.05 *m*/*z* [M + H]^+^.Anal. C_11_H_10_N_3_OCl (C, H, N).

**(8-Chloro-4-methyl-4**,**5-dihydropyrazolo[1**,**5-a]quinazoline-3-yl)methanol (15).** From **8** after 36 h; white crystals, recrystallized by ethanol 96%, yield 45%; mp 131–134 °C. TLC: toluene/ethyl acetate/methanol 8:2:1,5 *v*/*v*/*v*; ^1^H-NMR (400 MHz, CDCl_3_) δ 7.78 (s, 1H, H-2); 7.49 (d, 1H, H-6, *J =* 8.0 Hz); 7.38–7.42 (m, 3H, H-9, H-7 and OH); 4.42 (s, 2H, CH_2_N); 3.82 (s, 2H, CH_2_O); 2.49 (s, 3H, NCH_3_). ^13^C-NMR (100 MHz, CDCl_3_) δ 144.52, 138.54, 137.21, 131.65, 127.61, 126.76, 126.21, 115.09, 104.54, 62.67, 55.33, 38.40. ESI-MS calcd for C_12_H_12_N_3_OCl (249.70); found: 251.06 *m*/*z* [M + H]^+^.Anal. C_12_H_12_N_3_OCl (C, H, N).

**General procedure for obtaining compounds 17**, **18.** To a solution of starting material **1** or **8** [10] (0.38 mmol) dissolved in 5 mL of THF abs., 0.08 g (3.03 mmoles) of lithium borohydride and 1.5 mL of methanol were rapidly added. The reaction was maintained at reflux temperature, then was worked up by adding water/HCl 1N (10/3 mL) and extracted with ethyl acetate. After it dried under anhydrous sodium sulphate, the evaporation of the organic layer gave a residue recovered with water and filtered.

**8-Chloro-3-(hydroxymethyl)pyrazolo[1**,**5-a]quinazoline-5(4H)-one (17).** From **1**; white crystals, recrystallized by ethanol 96%, yield 55%; mp 117–119 °C. TLC: toluene/ethyl acetate/methanol 8:2:1.5 *v*/*v*/*v*; IR ν cm^−1^ 3100, 1718; ^1^H-NMR (400 MHz, DMSO-d_6_) δ 12.23 (exch br s, 1H, NH); 8.09 (d, 1H, H-6, *J =* 8.4 Hz); 8.00 (s, 1H, H-9); 7.78 (s, 1H, H-2); 7.49 (d, 1H, H-7, *J =* 8.4 Hz); 4.79 (m, 1H, OH, exch.); 4.33 (s, 2H, CH_2_O). ^13^C-NMR (100 MHz, DMSO-d_6_) δ 163.54, 151.41, 138.98, 138.85, 136.74, 126.67, 125.33, 123.31, 115.57, 104.34, 48.35. ESI-MS calcd for C_11_H_8_N_3_O_2_Cl (249.65); found: 251.03 *m*/*z* [M + H]^+^. Anal. C_11_H_8_N_3_O_2_Cl (C, H, N).

**8-Chloro-3-hydroxymethyl-4-methylpyrazolo[1**,**5-a]quinazoline-5(4H)-one (18).** From **8**; white crystals, recrystallized by ethanol 96%, yield 25%; mp 118–120 °C. TLC: toluene/ethyl acetate/methanol 8:2:1,5 *v*/*v*/*v*; ^1^H-NMR (400 MHz, DMSO-d_6_) δ 8.15 (d, 1H, H-6*, J =* 8.4 Hz); 8.03 (s, 1H, H-9); 7.85 (s, 1H, H-2); 7.52 (d, 1H, H-7, *J =* 8.4 Hz); 5.18 (m, 1H, OH, exch.); 4.56 (d, 2H, CH_2_O, *J =* 3.6 Hz); 3.75 (s, 3H, NCH_3_). ^13^C-NMR (100 MHz, DMSO-d_6_) δ 157.94, 144.97, 140.00, 138.10, 137.91, 130.86, 125.99, 114.34, 114.23, 106.12, 53.76, 30.54. ESI-MS calcd for C_12_H_10_N_3_O_2_Cl (263.68); found: 265.04 *m*/*z* [M + H]^+^. Anal. C_12_H_10_N_3_O_2_Cl (C, H, N).

Another procedure to obtain compound **18** with better yield and starting from **21** (achieved in turn from **20**) is here reported: the starting material 8-chloro-4-methyl-5-oxo-4,5-dihydropyrazolo[1,5-a]quinazoline-3-carboxyaldeide **21** (100 mg, 0.38 mmoles) was dissolved in a mixture of THF abs./methanol (3 mL/2 mL). Sodium borohydride 15.8 mg (0.42 mmoles) solution was added, portion wise, to this, and the reaction was stirred for 30′. The final solution evaporated to dryness, gave the row derivative **17**, which was recovered with water, filtered under suction and recrystallized by ethanol 96%.

**8-Chloro-4-methylpyrazolo[1**,**5-a]quinazoline-5(4H)-one (20).** The acid **10** (150 mg, 0.54 mmoles) was decarboxylated by refluxing in HCl conc, 20 mL, for 3 h, monitoring the reaction by TLC. The final solution was cooled, and ice was added to favor the precipitation of the final compound, filtered under suction. White crystals, yield 87%; mp 200–202 °C. TLC: toluene/ethyl acetate 8:2 *v*/*v*; ^1^H-NMR (400 Hz, DMSO-d_6_) δ 8.15 (d, 1H, H-6, *J =* 8.4 Hz); 8.05 (s, 1H, H-9); 7.89 (s, 1H, H-2); 7.53 (d, 1H, H-7, *J =* 8.4 Hz); 6.27 (s, 1H, H-3); 3.49 (s, 3H, NCH_3_). ESI-MS calcd for C_11_H_8_N_3_OCl (233.66); found: 235.03 *m*/*z* [M + H]^+^. Anal. C_11_H_8_N_3_OCl (C, H, N).

**8-Chloro-4-methyl-5-oxo-4**,**5-dihydropyrazolo[1**,**5-a]quinazoline-3-carbaldehyde (21).** The starting material **20** (100 mg, 0.40 mmoles) was dissolved in acetic acid (4 mL) and HMTA (hexamethylentetramina, 210 mg, 1.5 mmoles) was added. The reaction was refluxed for 8 h, monitored by TLC. The final solution was cooled, and treatment with ice yielded a precipitate filtered under suction. White crystals, yield 89%; mp 244–246 °C. TLC: CHX/ethyl acetate 1:5 *v*/*v*; ^1^H-NMR (400 MHz, DMSO-d_6_) δ 10.04 (s, 1H, CHO); 8.42 (s, 1H, H-2); 8.21 (d, 1H, H-6, *J =* 8.4 Hz); 8.13 (s, 1H, H-9); 7.65 (d, 1H, H-7, *J =* 8.4 Hz); 3.84 (s, 3H, NCH_3_). ESI-MS calcd for C_12_H_8_N_3_O_2_Cl (261.67); found: 263.03 *m*/*z* [M + H]^+^. Anal. C_12_H_8_N_3_O_2_Cl (C, H, N).

**General procedure for obtaining compounds 16 and 19.** A mixture of DMSO abs. (2 mL) and KOH (58.0 mg, 10.4 mmoles) was stirred at room temperature until dissolution. The starting material, **14**, **15** or **17**, **18** (0.26 mmoles) and methyl iodide (0.60 mmoles) were rapidly added. After 20 min, the starting material disappeared, evaluated by TLC, and the reaction was quenched with ice. The resulting precipitate was filtered by suction and recrystallized by a suitable solvent.

**8-Chloro-3-methoxymethyl-4-methyl-4**,**5-dihydropyrazolo[1**,**5-a]quinazoline (16).** From **14** or **15**; white crystals, recrystallized by ethanol 96%, yield 80%; mp 180–182 °C. TLC: toluene/ethyl acetate 8:2 *v*/*v*; ^1^H-NMR (400 MHz, CD_3_CN-d_3_) δ 8.23 (s, 1H, H-2); 8.14 (d, 1H, H-6, *J =* 8.4 Hz); 8.06 (d, 1H, H-9, *J =* 1.6 Hz); 7.58 (dd, 1H, H-7, *J_1_ =* 8.8 Hz, *J_2_ =* 1.6 Hz); 3.77 (s, 10H, CH_2_N, NCH_3_, CH_2_O, OCH_3_); ^13^C-NMR (100 MHz, CDCl_3_) δ 144.52, 138.54, 137.21, 131.65, 127.61, 126.76, 126.21, 115.09, 104.54, 62.67, 55.33, 38.40. ESI-MS calcd for C_13_H_14_N_3_OCl (263.73); found: 265.08 *m*/*z* [M + H]^+^. Anal. C_13_H_14_N_3_OCl (C, H, N).

**8-Chloro-3-methoxymethyl-4-methyl-4**,**5-dihydropyrazolo[1**,**5-a]quinazoline-5(4H)-one (19).** From **17** or **18**; white crystals, recrystallized by ethanol 96%, yield 83%; mp 135–137 °C. TLC: toluene/ethyl acetate/methanol 8:2:2 *v*/*v*/*v*; ^1^H-NMR (400 MHz, CDCl_3_) δ 7.78 (s, 1H, H-2); 7.50 (d, 1H, H-6, *J =* 8.0 Hz); 7.42–7.38 (m, 2H, H-9 and H-7); 4.56 (s, 2H, CH_2_O); 3.83 (s, 3H, NCH_3_); 2.50 (s, 3H, OCH_3_). ^13^C-NMR (100 MHz, DMSO-d_6_) δ 157.50, 145.60, 139.51, 130.72, 125.99, 114.08, 102.29, 64.10, 57.13, 30.45. ESI-MS calcd for C_13_H_12_N_3_O_2_Cl (277.71); found: 279.06 *m*/*z* [M + H]^+^. Anal. C_13_H_12_N_3_O_2_Cl (C, H, N).

### 3.2. Molecular Docking and Molecular Dynamic Simulation

The structure of the binding site was obtained from the human α1β2γ2-GABA_A_ receptor subtype in complex with GABA and flumazenil, conformation B (PDB ID 6D6T) [3], considering all the amino acids within a distance of about 2 nm from the structure of the flumazenil. The ligands were placed at the binding site through AUTODOCK 4.2. [13]

The molecular dynamics simulations of ligand–binding site complexes were performed on a minimum number of conformations (maximum 2), to cover at least 90% of the poses found by AUTODOCK.

A 60 ns MD simulation was performed for all complexes using the GROMACS v5.1 program, and it was conducted in vacuum [14]. The DS ViewerPro 6.0 program [15] was used to build the initial conformations of ligands. The partial atomic charge of the ligand structures was calculated with CHIMERA [16] using the AM1-BCC method, and the topology was created with ACPYPE [17] based on the routine Antechamber [18].

The OPLS-AA/L all-atom force field [19] parameters were applied to all the structures. To remove bad contacts, energy minimization was performed using the steepest descent algorithm, until convergence was achieved, or for 50,000 maximum steps. The next equilibration of the system was conducted in two phases:

(1) canonical NVT ensemble, a 100 ps position restraint of molecules at 300 K was carried out using a temperature-coupling thermostat (velocity-rescaling, in stochastic terms) to ensure the proper stabilization of the temperature [20];

(2) isothermal isobaric NPT ensemble, a 100 ps position restraint of molecules at 300 K and 1 bar was carried out without using barostat pressure coupling to stabilize the system. These were then followed by a 60 ns MD run at 300 K with position restraints for all protein atoms. The Lincs algorithm [21] was used for bond constraints to maintain rigid bond lengths.

The initial velocity was randomly assigned taken from Maxwell–Boltzman distribution at 300 K and computed with a time step of 2 fs, and the coordinates were recorded every 0.6 ns for MD simulation of 60 ns. The conformations collected during the simulated trajectory were 100.

The ‘Proximity Frequencies’ (PFs) [9], with which the 100 conformations of each binding site–ligand complex intercepts two or more amino acids during the dynamic simulation, were calculated. The ‘Proximity Frequency’ (PF) is the frequency with which the ligand was, during the molecular dynamic simulation, at a distance of less than 0.25 nm from an amino acid of the binding site and also, simultaneously, from 2, 3 and 4 amino acids of the binding site.

### 3.3. Biological Experimental

#### 3.3.1. Expression of Human Receptor Subunits

A mixture of pCDM8-based vectors for the α_1_, β_2_ or γ_2L_ subunits of human GABA_A_ receptors (total of 1.5 ng of DNA, comprising equal amounts of α, β and γ subunit vectors), or an equal amount of α and β receptors for the expression of α_1_β_2_ receptors, were injected into the animal pole of *X. laevis* oocytes, as described [22], with the use of a microdispenser (Drummond Scientific, Broomwall, PA, USA). The injected oocytes were maintained at 13 °C in sterile modified Barth’s solution [MBS: 88 mM NaCl, 1 mM KCl, 10 mM HEPES-NaOH (pH 7.5), 0.82 mM MgSO_4_, 2.4 mM NaHCO_3_, 0.91 mM CaCl_2_, 0.33 mM Ca(NO_3_)_2_] supplemented with streptomycin (10 mg/L), penicillin (10,000 U/L), gentamicin (50 mg/L), theophylline (90 mg/L) and pyruvate (220 mg/L).

#### 3.3.2. Electrophysiology

Electrophysiological measurements were performed in oocytes 2 to 4 days after DNA injection. Oocytes were placed in a rectangular chamber (volume~100 µL) and perfused at a rate of 1.7 mL/min with MBS at room temperature with the use of a roller pump (Cole-Parmer, Chicago, IL, USA) and 18-gauge polyethylene tubing (Clay Adams, Parsippany, NJ, USA). Oocytes were impaled at the animal pole with two glass electrodes (0.5 to 10 MΩ) filled with 3 M KCl and were clamped at –70 mV with the use of an oocyte clamp (model OC725C; Warner Instruments, Hamden, CT). GABA-induced Cl- currents were measured and analyzed with the pClamp 9.2 software (Molecular Devices, Union City, CA, USA). GABA (Sigma, St. Louis, MO, USA) was dissolved in MBS and applied to the oocytes for 30 s. Oocytes were perfused with test drugs for 30 s either in the absence of the GABA or in its presence at the EC5-10 (the concentration of agonist that induces a peak current equal to 5 to 10% of the maximal current elicited by the maximal concentration of the agonist). The EC5 concentration was determined for each oocyte and was approximately 3–5 μM [23]. Experimental sequence was as follows: maximal GABA (1 mM GABA, 20 s application, 20 min. washout); EC5-10 GABA (30 s application, 10/15 min washout), pre-application of the drug (30 s); followed by a co-application with EC10 GABA, EC10 GABA (30 s). Test drugs were first dissolved in DMSO at a concentration of 10 mM and then diluted in MBS to the final concentrations. In each experiment, control responses were determined before and 10/15 min after application of the drug.

#### 3.3.3. Statistic

Statistical analysis was performed on normalized data using the one-way ANOVA test followed by Dunn’s post hoc test using Graph Pad Prism 9 (Graph Pad Software, Inc., San Diego, CA, USA).

## 4. Conclusions

The synthesis of 8-chloropyrazolo[1,5-a]quinazolines and 8-chloro-4,5-dihydropyrazolo[1,5-a]quinazolines was designed and realized and all new compounds (**3**, **4**, **6a**–**c**, **7a**–**b**, **8**, **9**, **12a**–**c**, **13a**,**b**, **14**–**19**) were subjected to a molecular dynamic study performed on an isolated portion of the GABA_A_ receptor protein, between the α and γ chains, where the benzodiazepine binding site is recognized. Then, applying the ‘Proximity Frequencies’ model (PF) [9], we obtained a prediction that collocates **6a**, **13a** and **18** in the agonist class, reaching a percentage of prediction of 93.1%. Compounds **3**, **9** and **19** are collocated in the antagonist class, with a percentage prediction range of 62–73%. Interestingly, these two classes of compounds occupy different areas in the binding site that might justify the different predicted profile. The virtual prediction for **18** and **19** as agonist and antagonist, respectively, was confirmed through electrophysiological assays: compound **18** significantly enhances the chlorine current at 100 μM (E_max_ + 100%), and it was antagonized by flumazenil (100 µM), thus acting as an agonist, while compound **19** was able to antagonize the chlorine current produced by the standard agonist lorazepam, thus confirming its antagonist profile. In conclusion, our PF model can be a useful predictive model of the efficacy/profile of new benzodiazepine site ligands.

## Figures and Tables

**Table 1:** Prediction obtained by PF model ^a^.

N°	Pred	Ago%	Anta%
3	anta	27.1	72.9
4	anta	14.7	85.3
4	ago	64.2	35.8
6a	ago	63.5	36.5
6a	ago	93.1	6.9
6b	ago	52.4	47.6
6b	ago	57.3	42.7
6c	anta	43.2	56.8
6c	ago	63.5	36.5
7a	ago	63.5	36.5
7a	ago	62.0	38.0
7b	ago	62.9	37.1
7b	anta	49.1	50.9
8	anta	40.6	59.4
8	anta	37.9	62.1
9	anta	12.7	87.3
12a	anta	46.1	53.9
12a	ago	70.5	29.5
12a	ago	63.5	36.5
12b	ago	64.2	35.8
12b	ago	63.5	36.5
12c	ago	63.5	36.5
13a	ago	72.4	27.6
13b	ago	58.3	41.7
13b	ago	54.2	45.8
14	ago	54.2	45.8
14	ago	50.2	49.8
15	anta	46.0	54.0
16	anta	48.7	51.3
17	ago	63.5	36.5
18	ago	63.5	36.5
18	ago	64.8	35.2
19	anta	37.9	62.1

^a^ In red, predicted antagonists; in green, predicted agonists.

## Data Availability

Not applicable.

## Supplementary Materials

The following supporting information can be downloaded at: https://www.mdpi.com/article/10.3390/ijms232113032/s1.
