# Supplementary material for: GABAA Receptor Modulators with a Pyrazolo[1,5-a]quinazoline Core: Synthesis, Molecular Modelling Studies and Electrophysiological Assays
Source: Int J Mol Sci. 2022 Oct 27;23(21):13032. doi: 10.3390/ijms232113032 (PMC9658275; doi:10.3390/ijms232113032)

Supporting Information

for

GABA$_A$ Receptor Modulators with a Pyrazolo[1,5-a]quinazoline Core: Synthesis, Molecular Modelling Studies and Electrophysiological Assays

Letizia Crocetti [1], Gabriella Guerrini [1]*, Fabrizio Melani [1], Claudia Vergelli[1], Maria Paola Mascia[2] and Maria Paola Giovannoni[1]

[1]Neurofarba, Pharmaceutical and Nutraceutical Section, University of Florence, Via Ugo Schiff 6, 50019, Sesto Fiorentino, Italy.

[2]CNR-Institute of Neuroscience, Cagliari, Cittadella Universitaria, 09042, Monserrato, Italy.

*Correspondence: gabriella.guerrini@unifi.it; Tel.: +39-055-4573766

Table of contents:

- NMR spectra of some representative compounds (**1-3, 5, 6a, 6c, 8, 12a-c, 14, 16-21**)

1H NMR Compound 1
DMSO

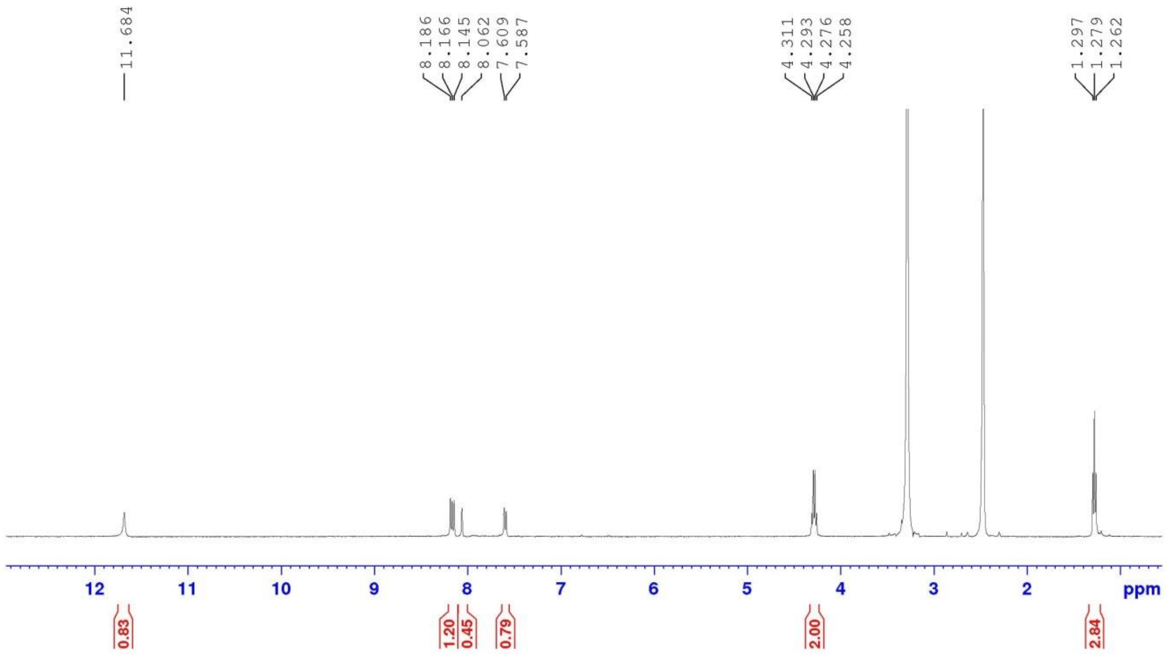

1H NMR Compound 2
DMSO

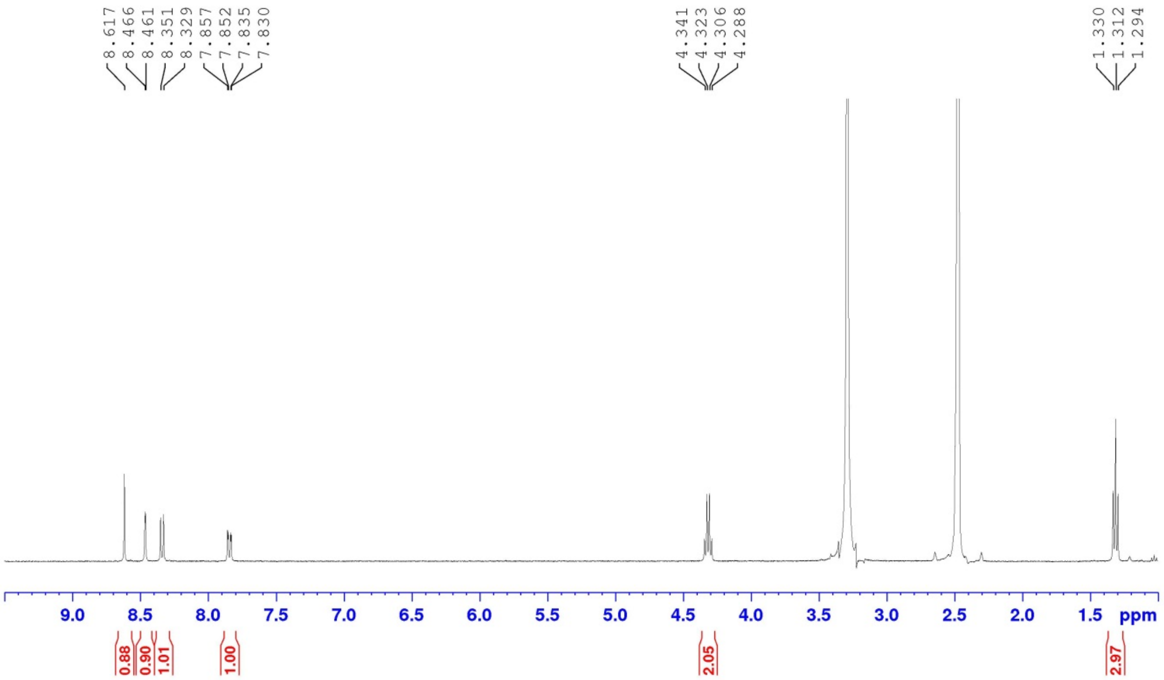

1H NMR Compound 3
CDCl3

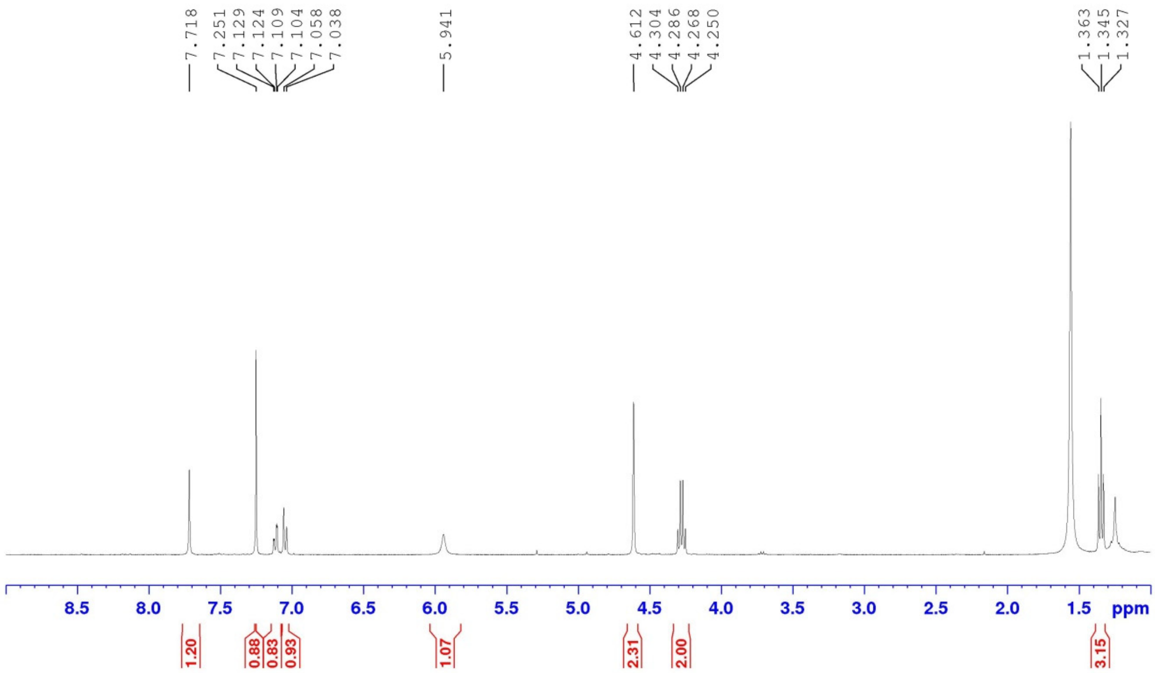

13C NMR Compound 3
DMSO

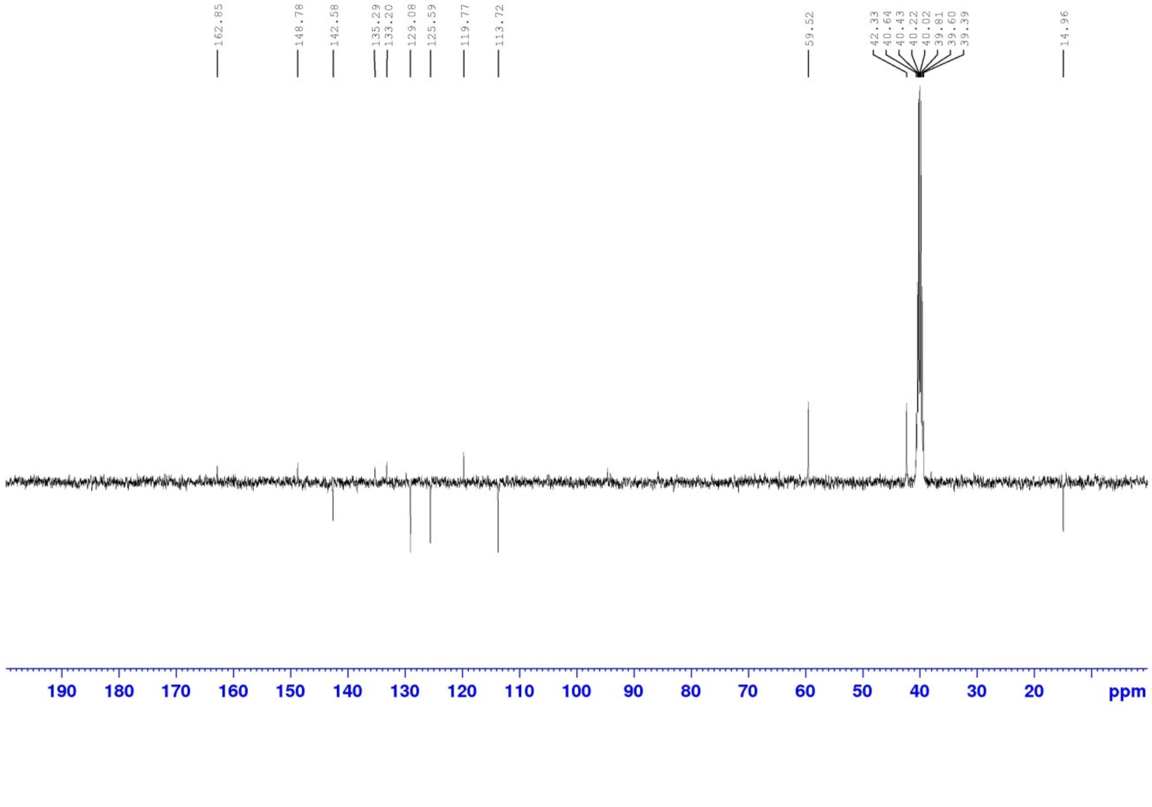

1H NMR Compound 5
DMSO

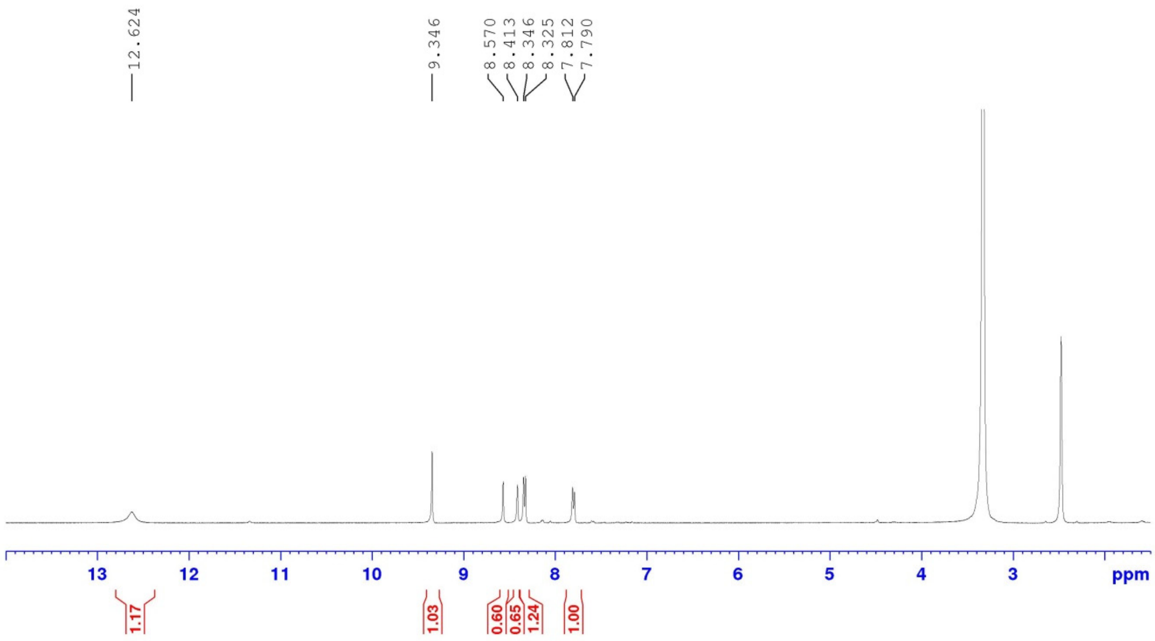

1H NMR Compound 6a
DMSO

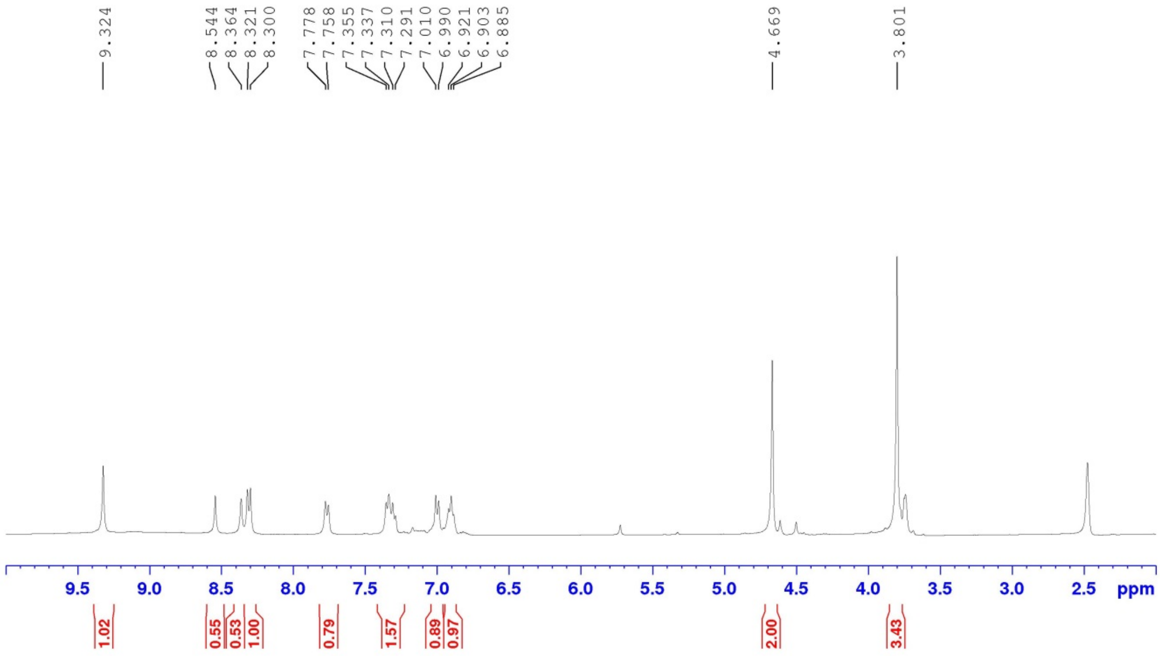

13C NMR Compound 6a
DMSO

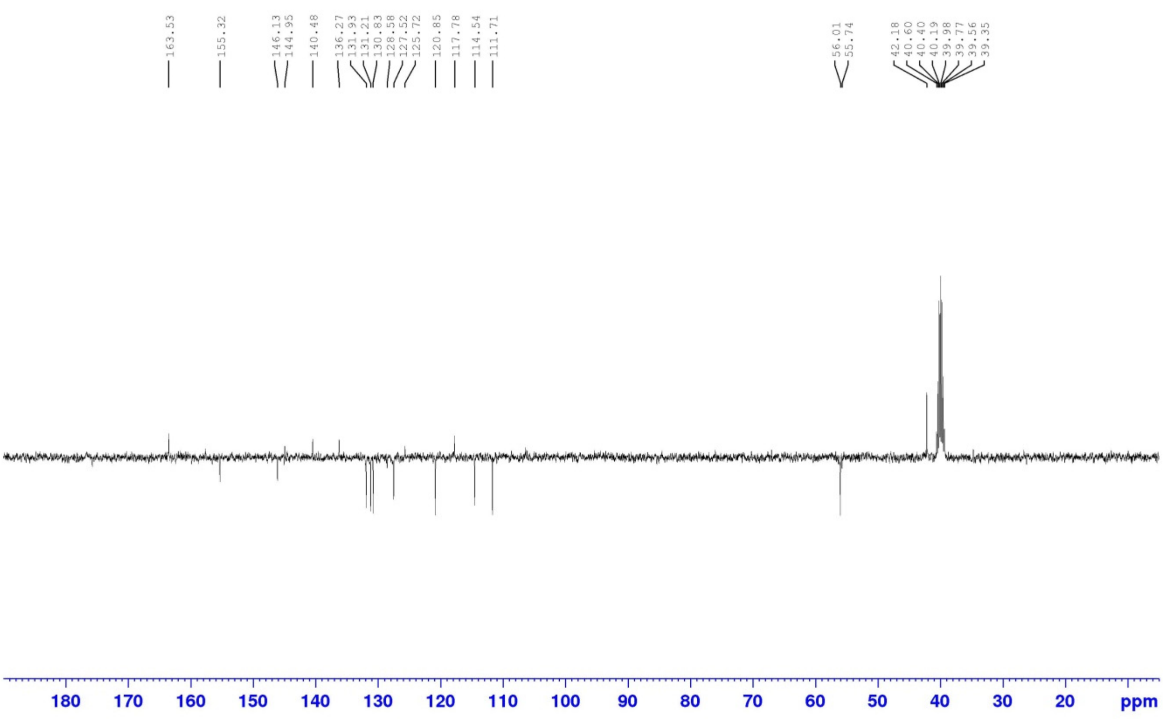

1H NMR Compound 6c
DMSO

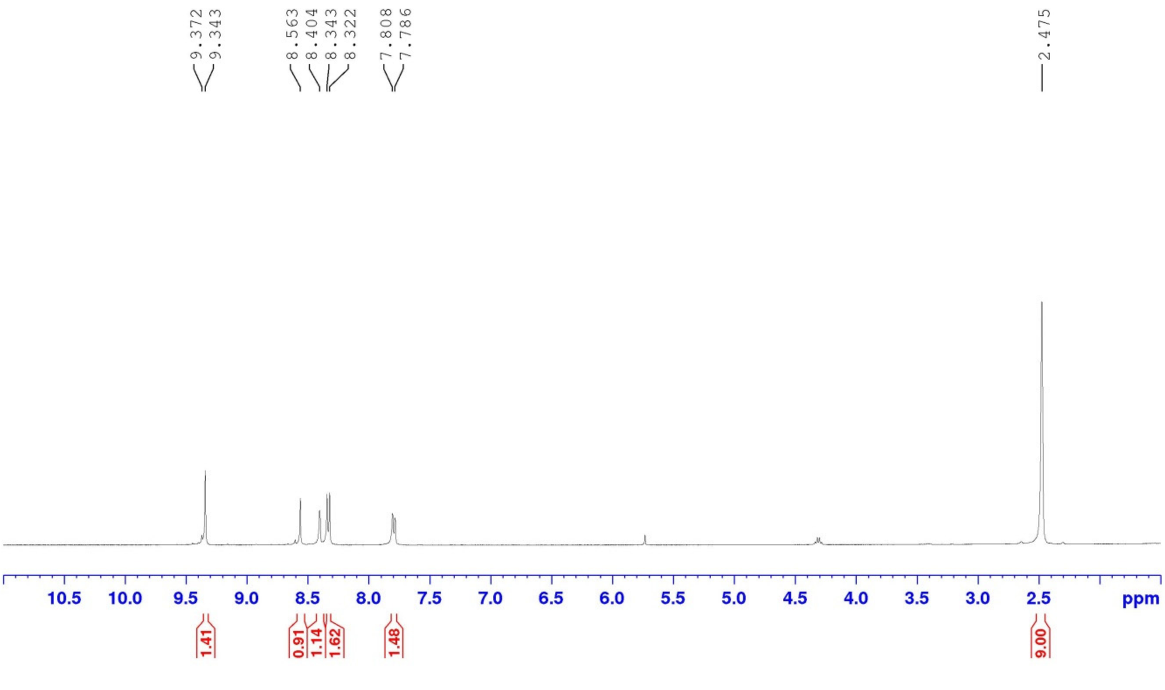

13C NMR Compound 6c
DMSO

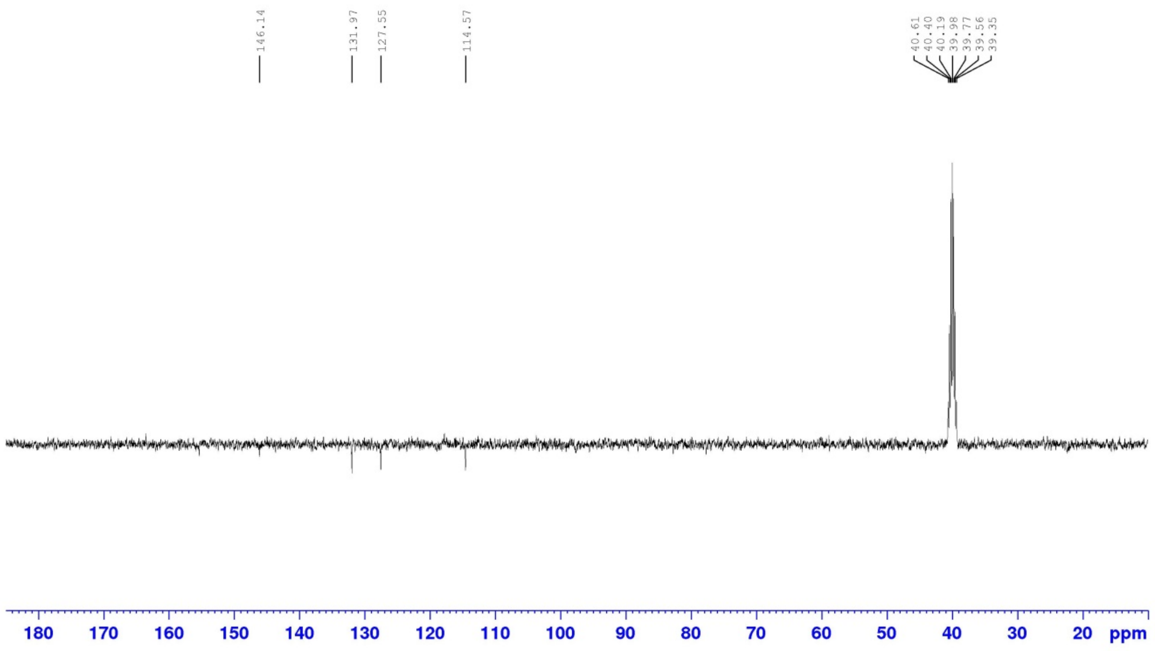

1H NMR Compound 8
DMSO

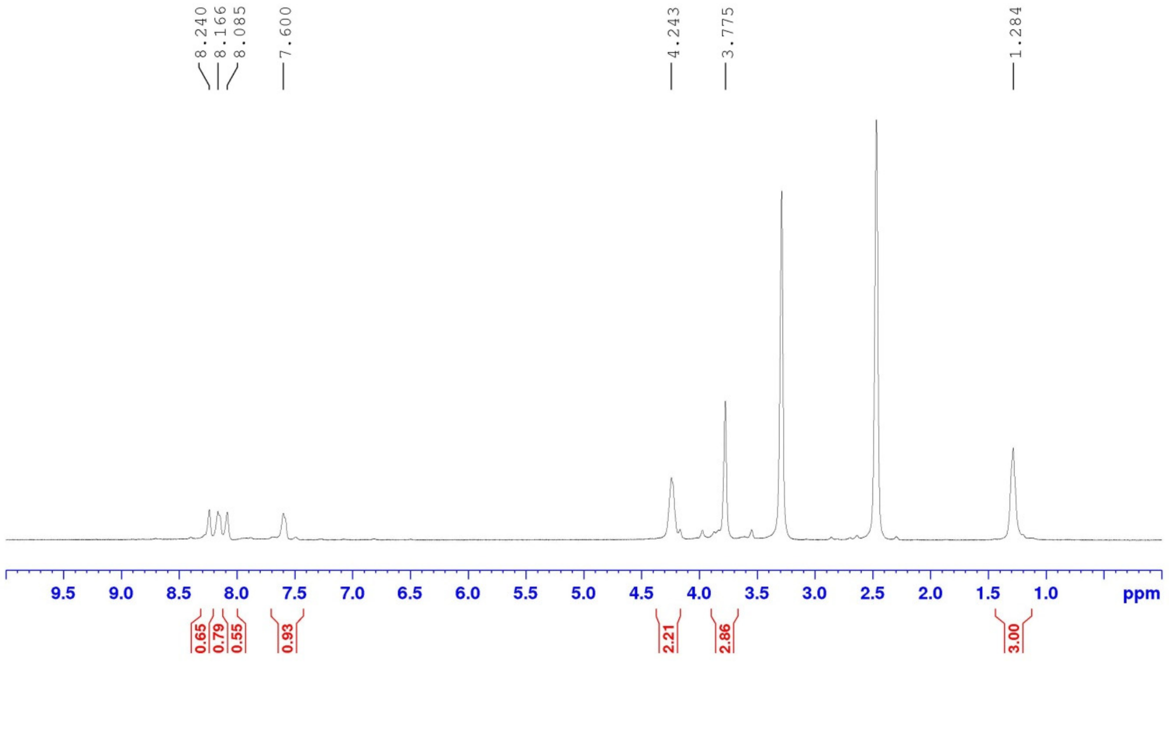

13C NMR Compound 8
DMSO

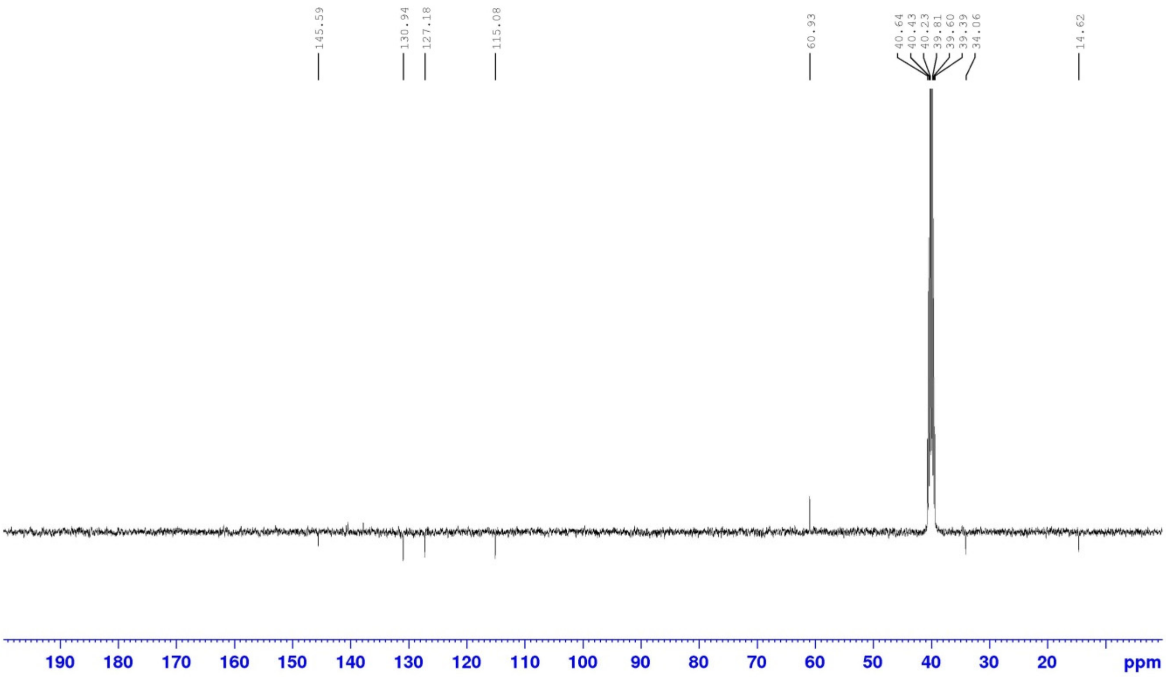

1H NMR Compound 12a
DMSO

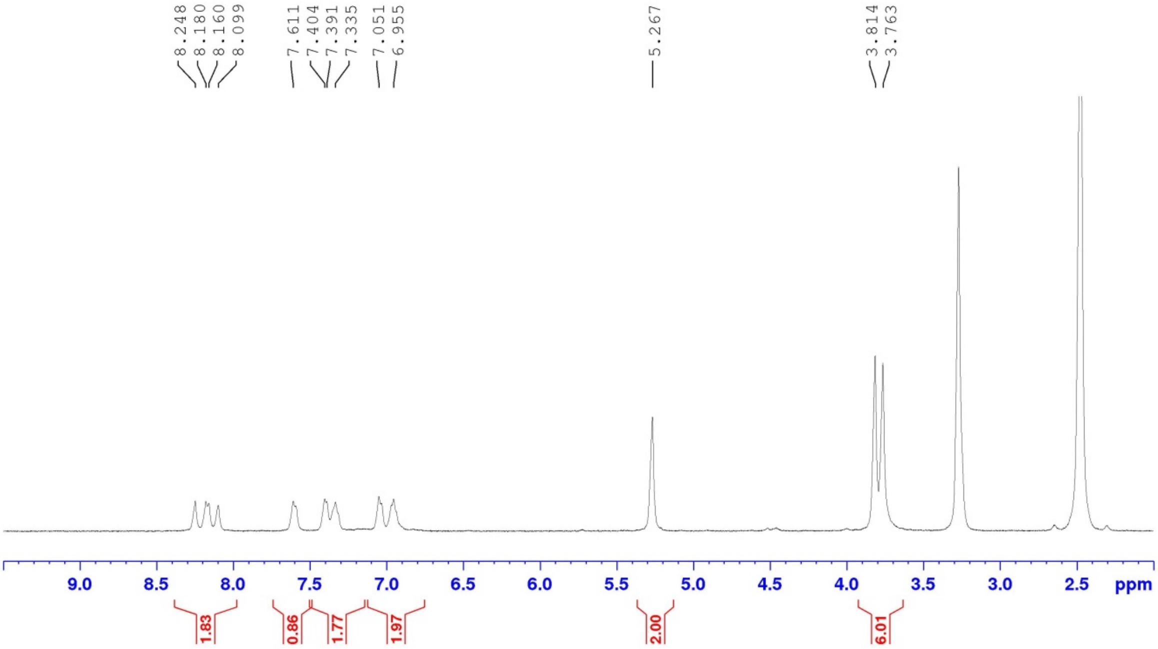

13C NMR Compound 12a
DMSO

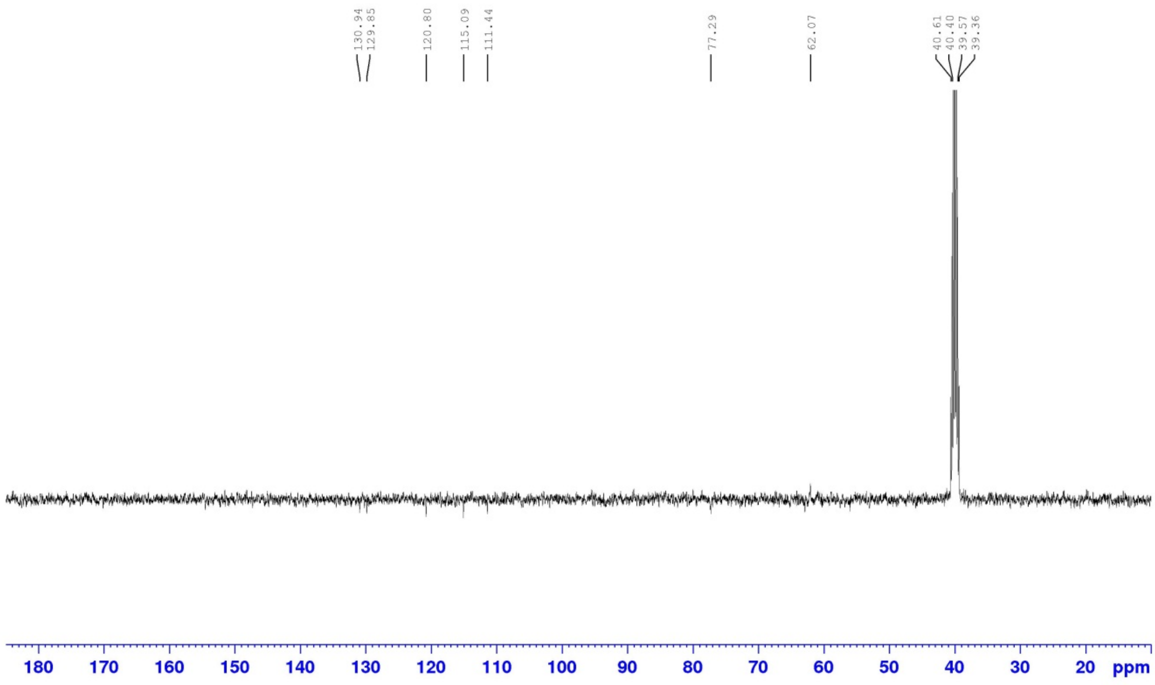

1H NMR Compound 12b
DMSO

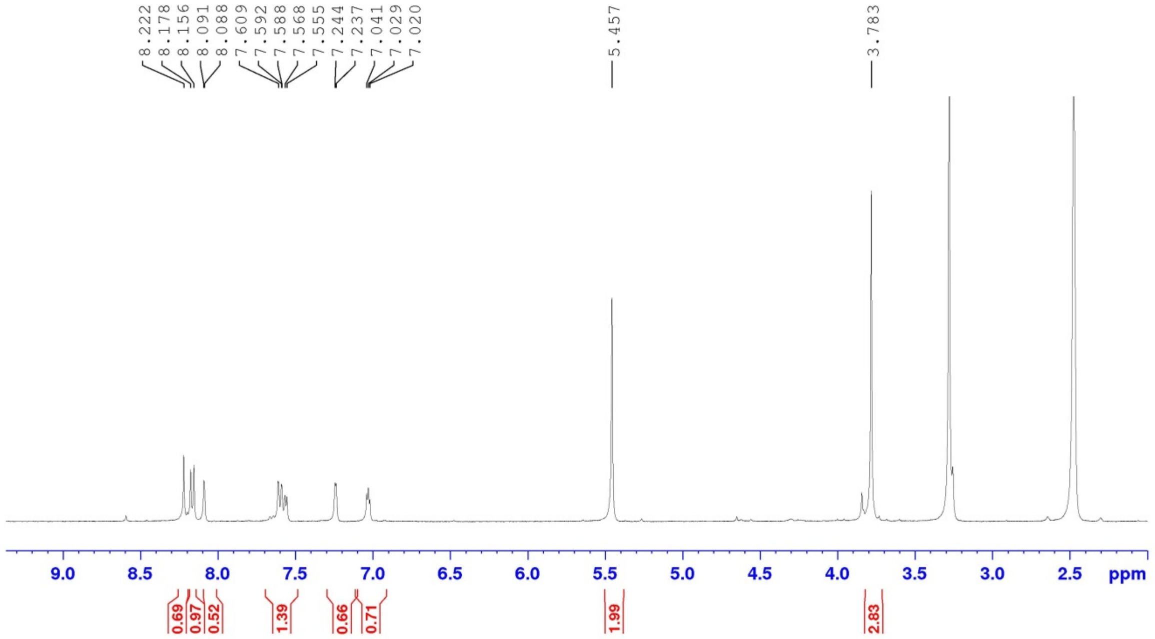

13C NMR Compound 12b
DMSO

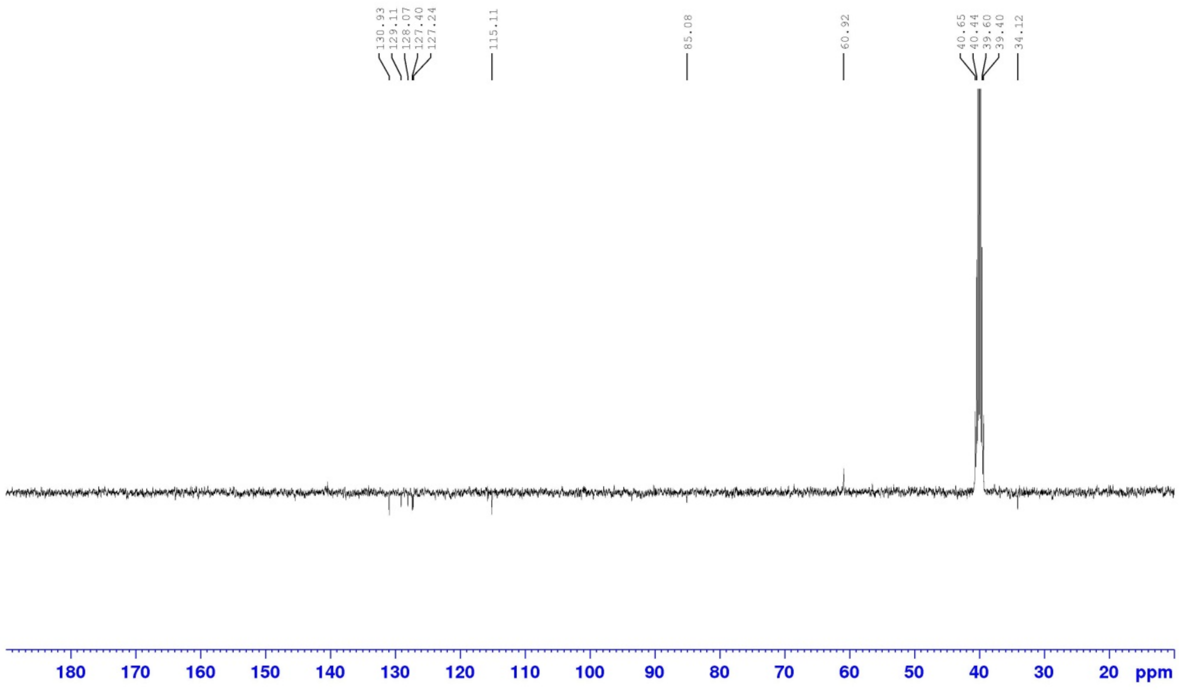

1H NMR Compound 12c
DMSO

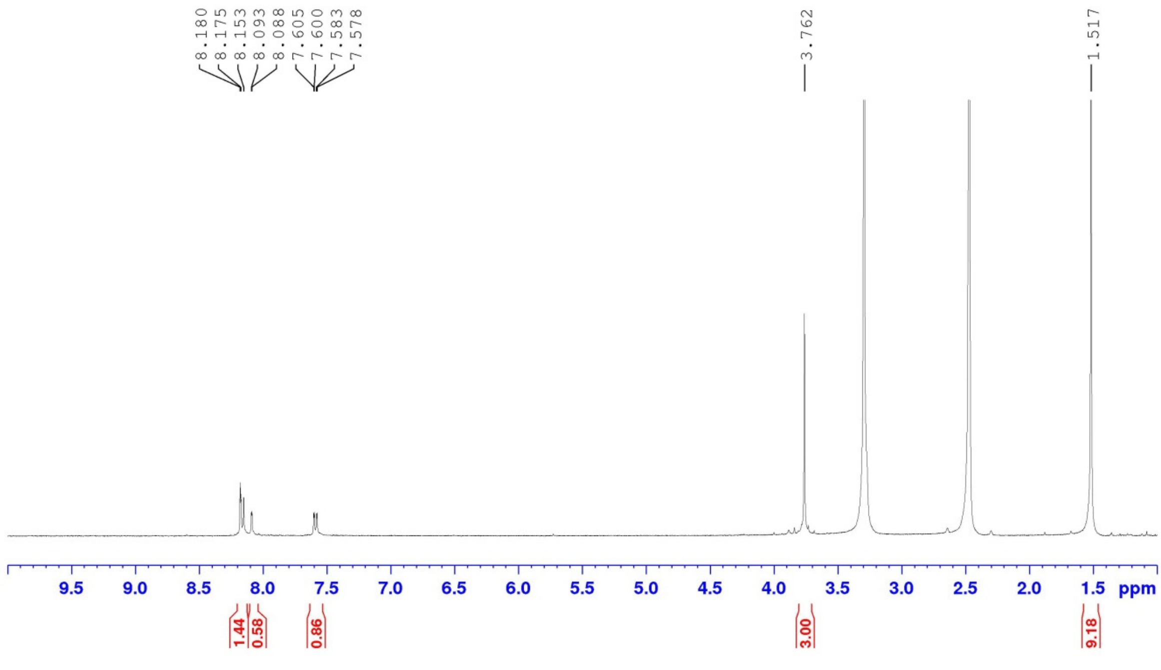

1H NMR Compound 14
DMSO

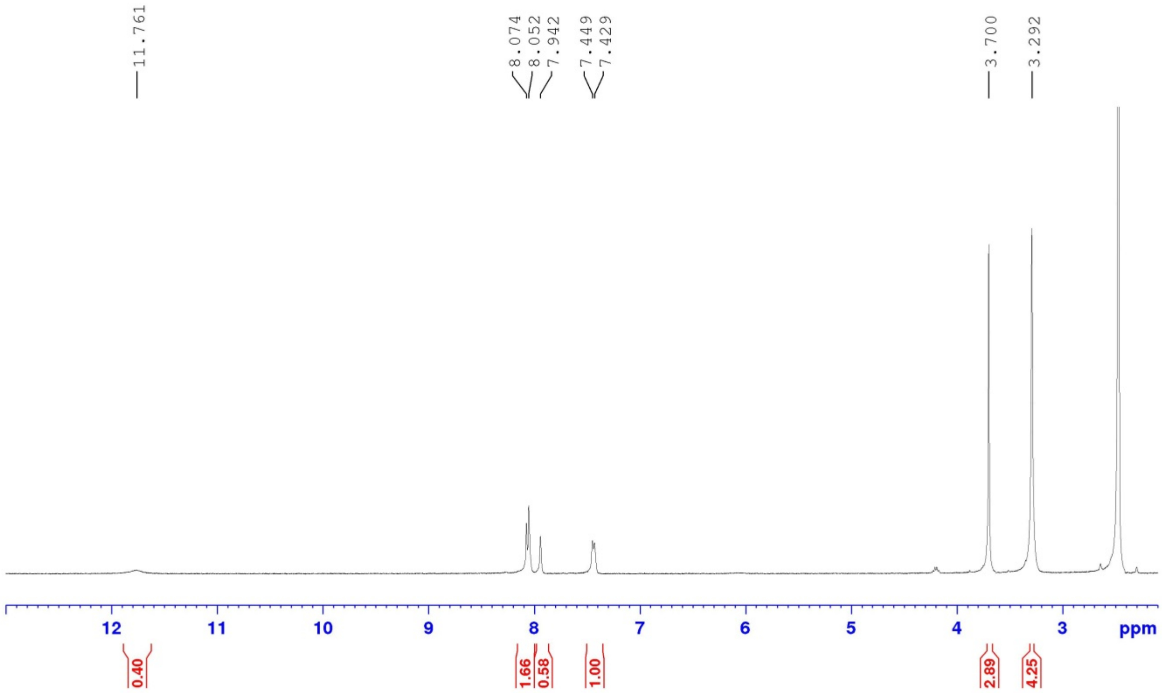

1H NMR Compound 16
DMSO

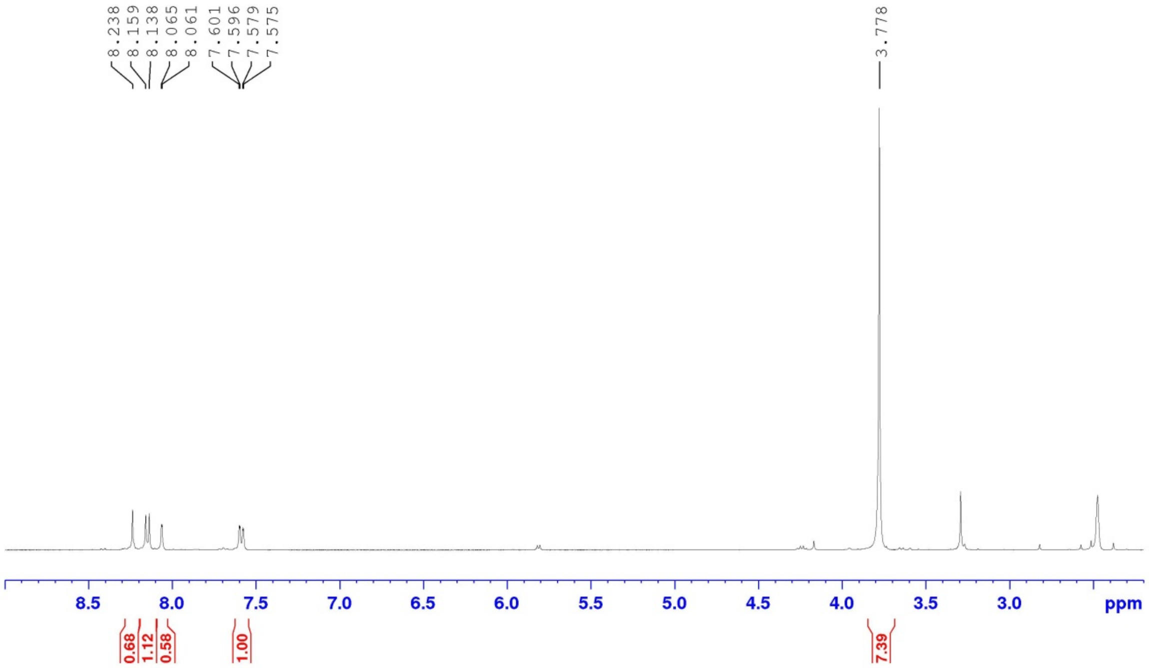

1H NMR Compound 17
DMSO

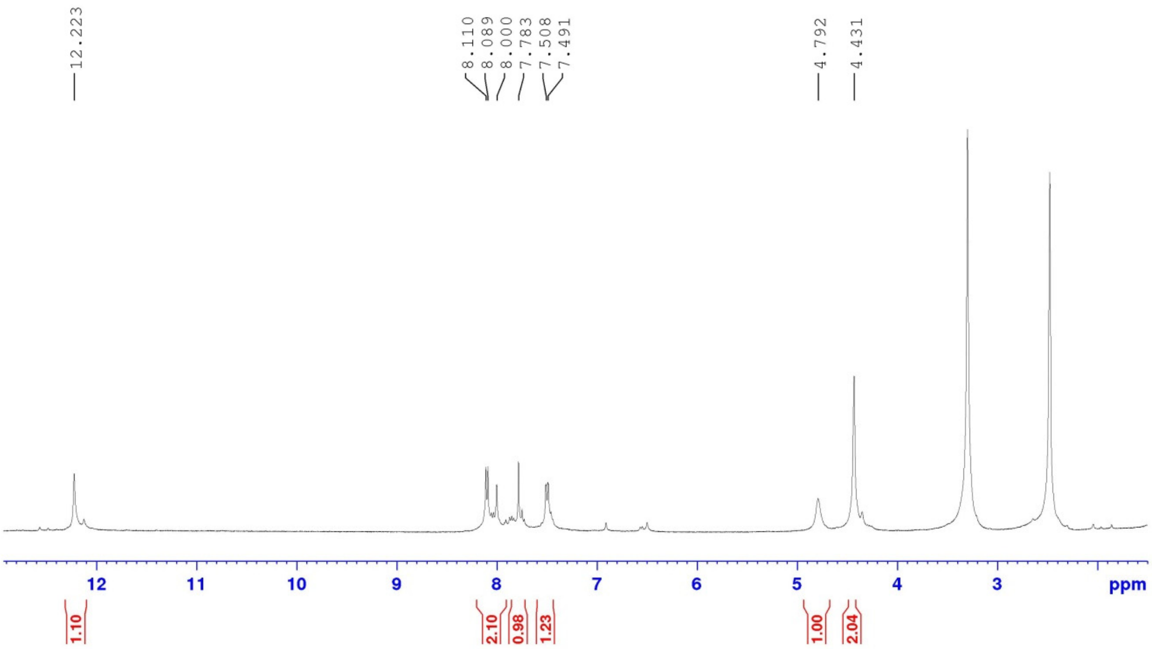

1H NMR Compound 18
DMSO

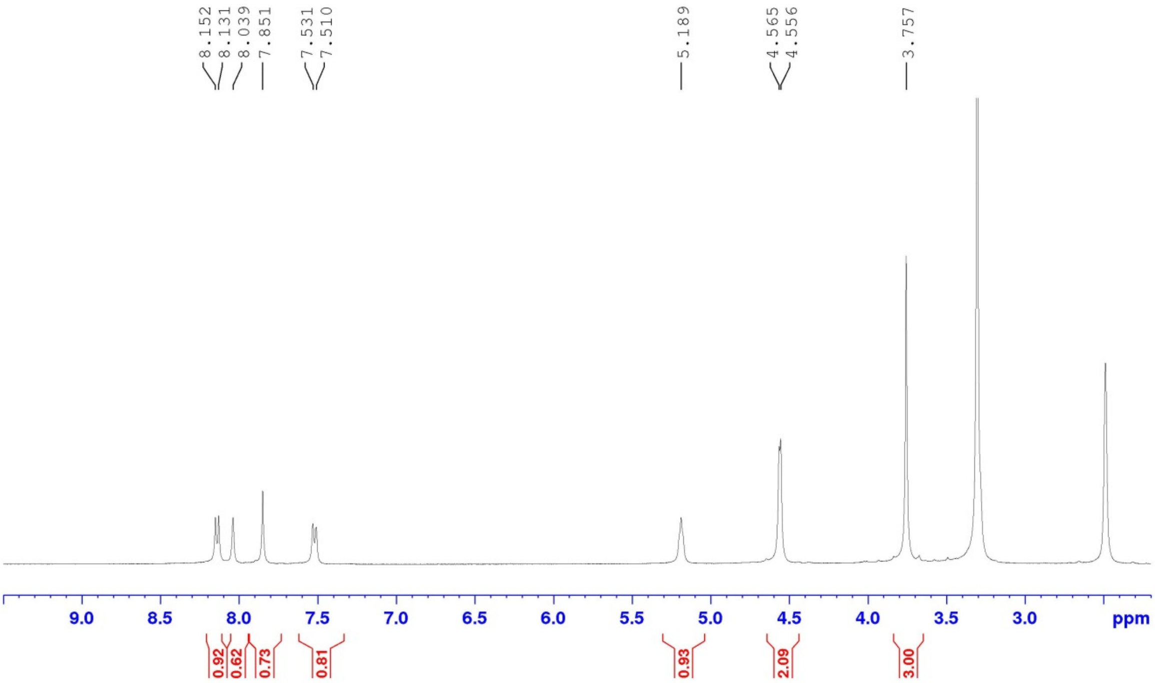

13C NMR Compound 18
DMSO

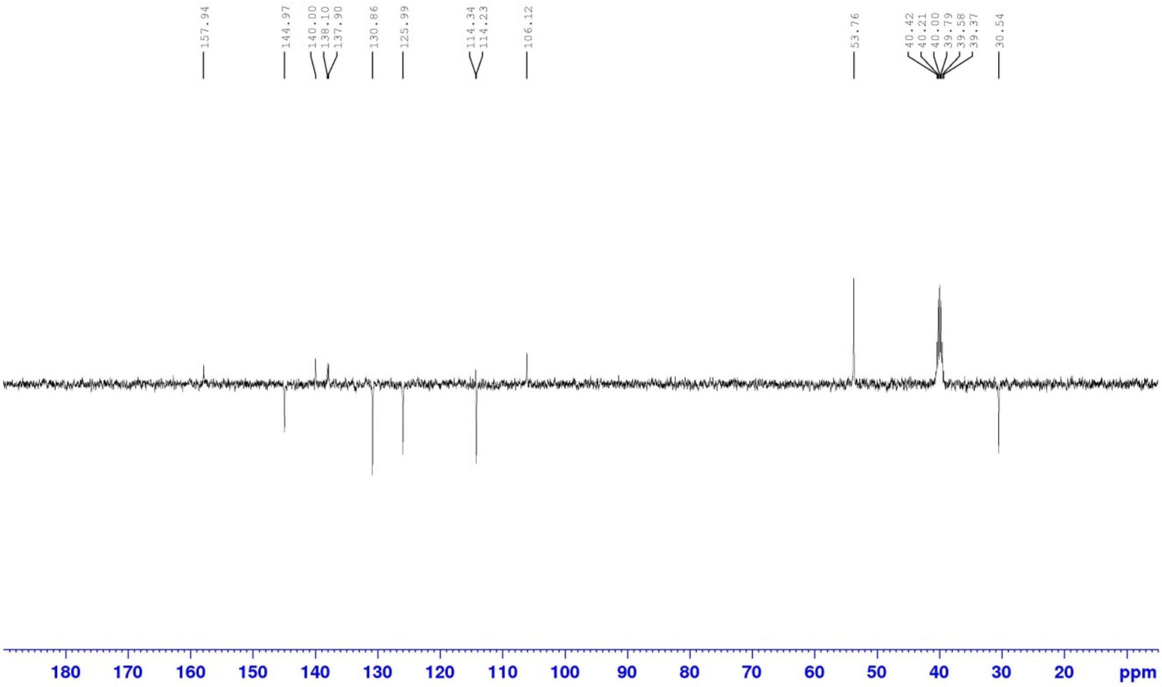

1H NMR Compound 19
DMSO

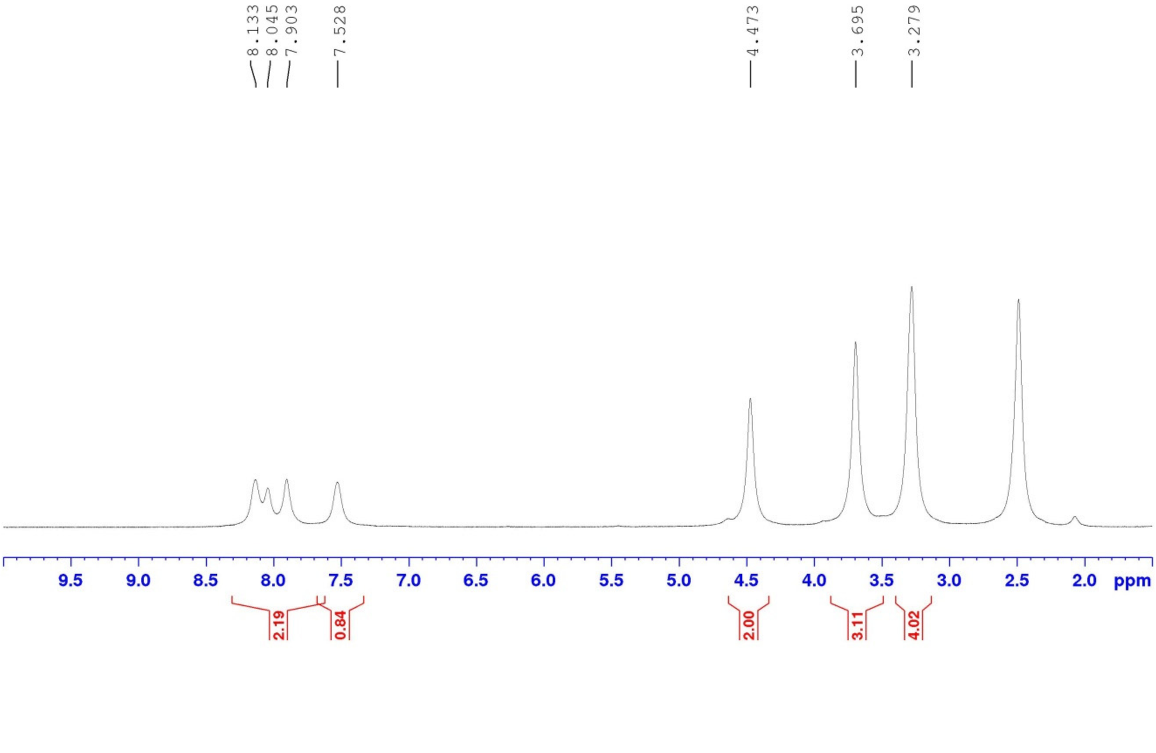

13C NMR Compound 19
DMSO

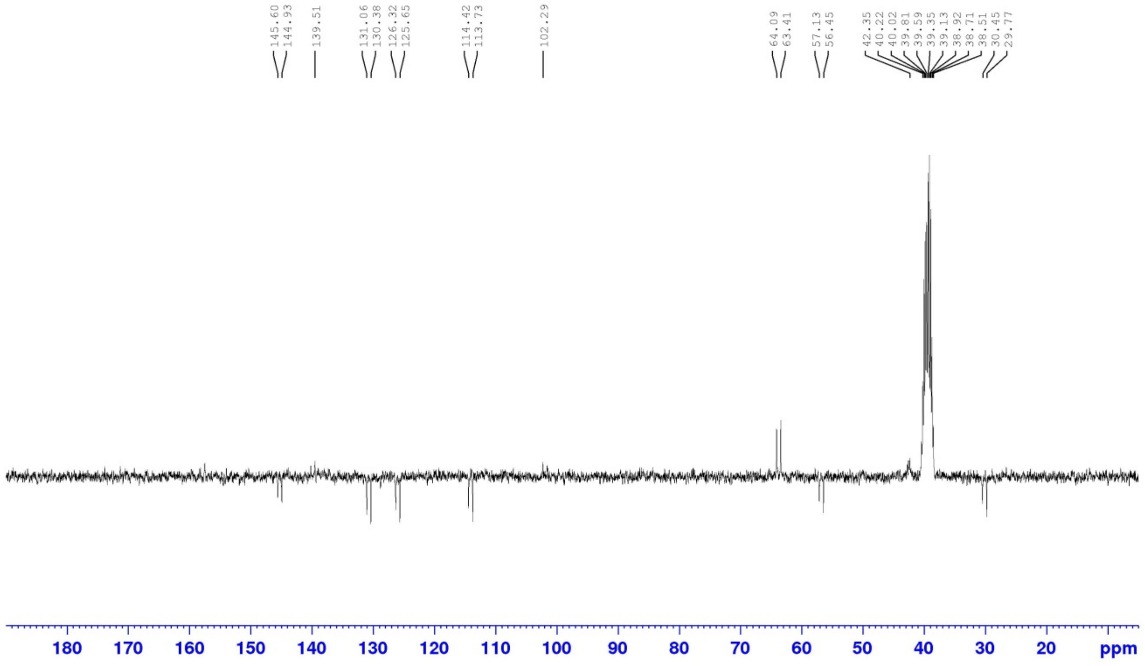

1H NMR Compound 20
DMSO

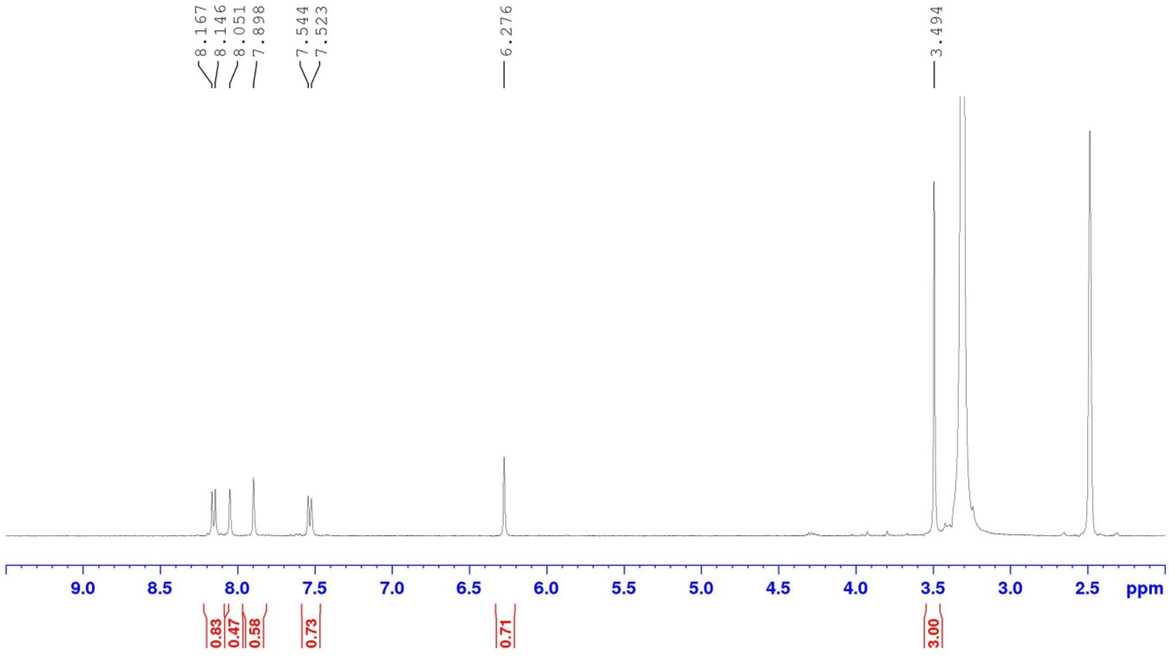

1H NMR Compound 21
DMSO

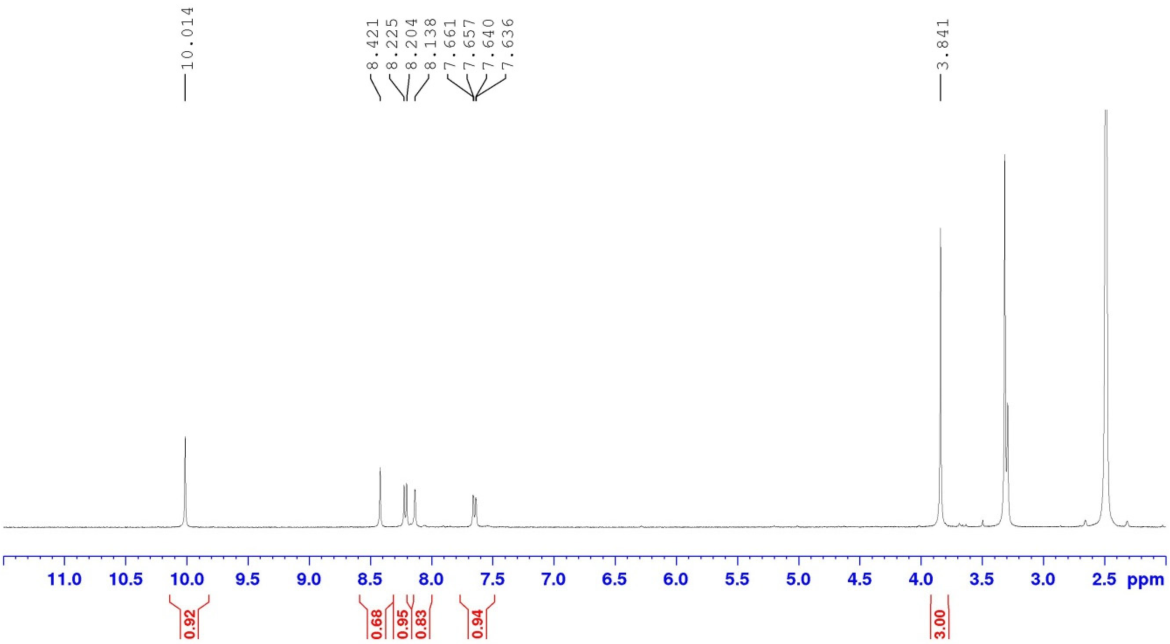

13C NMR Compound 21
DMSO

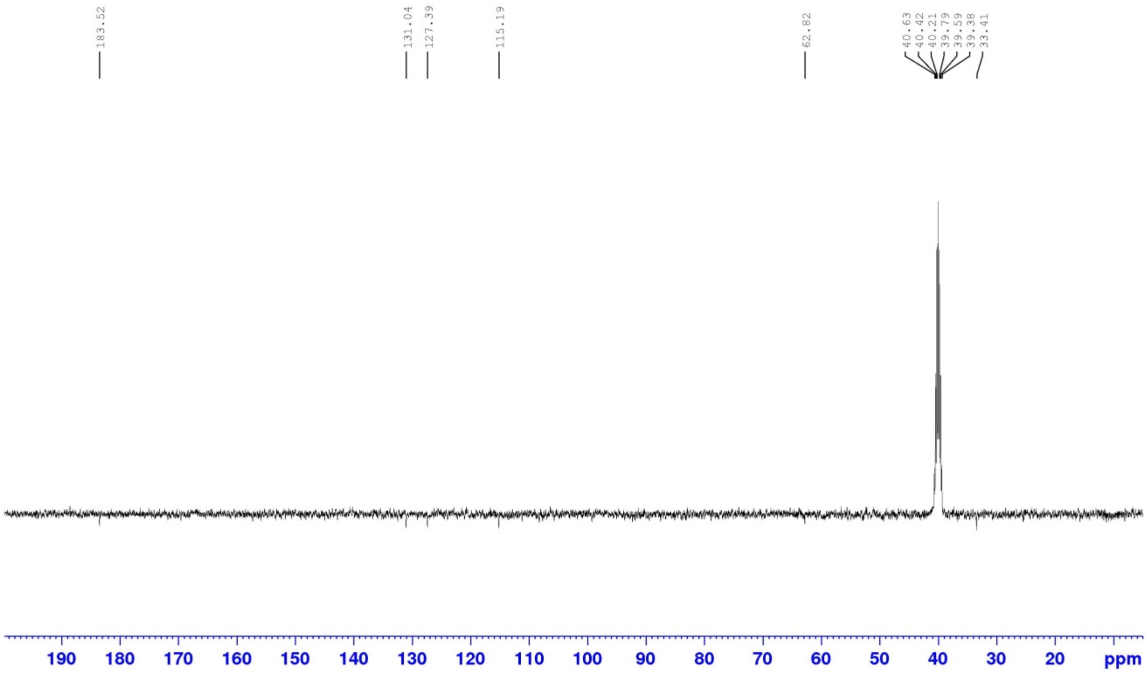

Supplement: Supplementary file 1 [file ijms-23-13032-s001.zip › ijms-1978613-supplementary.pdf]
